# Decavanadate Inhibits Mycobacterial Growth More Potently Than Other Oxovanadates

**DOI:** 10.3389/fchem.2018.00519

**Published:** 2018-11-20

**Authors:** Nuttaporn Samart, Zeyad Arhouma, Santosh Kumar, Heide A. Murakami, Dean C. Crick, Debbie C. Crans

**Affiliations:** ^1^Department of Chemistry, Colorado State University, Fort Collins, CO, United States; ^2^Department of Chemistry, Rajabhat Rajanagarindra University, Chachoengsao, Thailand; ^3^Cell and Molecular Biology Program, Colorado State University, Fort Collins, CO, United States; ^4^Department of Microbiology, Immunology and Pathology, Colorado State University, Fort Collins, CO, United States

**Keywords:** vanadate, speciation, sodium metavanadate, decavanadate, growth inhibition, tuberculosis

## Abstract

^51^V NMR spectroscopy is used to document, using speciation analysis, that one oxometalate is a more potent growth inhibitor of two Mycobacterial strains than other oxovanadates, thus demonstrating selectivity in its interaction with cells. Historically, oxometalates have had many applications in biological and medical studies, including study of the phase-problem in X-ray crystallography of the ribosome. The effect of different vanadate salts on the growth of *Mycobacterium smegmatis* (*M. smeg*) and *Mycobacterium tuberculosis* (*M. tb*) was investigated, and speciation was found to be critical for the observed growth inhibition. Specifically, the large orange-colored sodium decavanadate (V_10_${\text{O}}_{28}^{6-}$) anion was found to be a stronger inhibitor of growth of two mycobacterial species than the colorless oxovanadate prepared from sodium metavanadate. The vanadium(V) speciation in the growth media and conversion among species under growth conditions was monitored using ^51^V NMR spectroscopy and speciation calculations. The findings presented in this work is particularly important in considering the many applications of polyoxometalates in biological and medical studies, such as the investigation of the phase-problem in X-ray crystallography for the ribosome. The findings presented in this work investigate the interactions of oxometalates with other biological systems.

## Introduction

Polyoxometalates (POMs) are a class of compounds that have been investigated in a range of biological and biomedical systems, as their effects in cell culture studies and *in vivo* suggest that these compounds have potential for use as therapeutic agents (Moskovitz and Group, 1988; Hill et al., 1990; Rhule et al., 1998; Aureliano and Crans, 2009; Fraqueza et al., 2012; Aureliano et al., 2013, 2016; Wang et al., 2013; Aureliano and Ohlin, 2014; Leon et al., 2014; Kioseoglou et al., 2015; Shah et al., 2015; Sun et al., 2016; Fu et al., 2018; Gumerova et al., 2018). Decavanadate is a homopolyoxometalate anion, and one of the POMs that has been reported to have known biological effects, as documented by studies reported with cells as well as with isolated enzyme systems. (Pluskey et al., 1996; Aureliano and Crans, 2009; Zhai et al., 2009; Fraqueza et al., 2012; Turner et al., 2012; Aureliano et al., 2013; Kioseoglou et al., 2013; Aureliano, 2014, 2016; Aureliano and Ohlin, 2014). Protein crystal structures have been reported for some protein-POM complexes such as those reported between the ribosome and a Dawson oxometallate (Weinstein et al., 1999; Auerbach-Nevo et al., 2005; Bashan and Yonath, 2008; Noeske et al., 2015). Other protein-POM complexes include protein complexes with smaller oxometalates such as decavanadate (Winkler et al., 2017). The limited stability of decavanadate at neutral pH would suggest that hydrolysis intermediates may form and generate stable complexes with proteins or cellular components. Speciation studies are important in this regard, and different species and possibilities must be considered when investigating the mode of action of systems that are not thermodynamically stable (Aureliano and Crans, 2009; Levina et al., 2017a). Even if the speciation is characterized, the active species and mode of action of these complex systems can be non-trivial to interpret (Willsky et al., 1984b, 1985, 2011; Delgado et al., 2005; Crans et al., 2011; Postal et al., 2016; Jakusch and Kiss, 2017). However, a wide range of activities have been reported depending on the protein, biological system or specific vanadium species (Crans, 2000; Crans et al., 2013; Correia et al., 2015; Postal et al., 2016). Recently, it has become clear that compound uptake is critical to the mode of action because many vanadium compounds are modified during the uptake process (Pessoa and Tomaz, 2010; Crans et al., 2011; Le et al., 2017; Levina et al., 2017a). In the case of a large anion such as decavanadate, the question is simply whether the species is too large to enter through protein channels and thus must be taken up through endocytosis or passive transport mechanisms. The alternative possibility is that the uptake is of the smaller vanadium oxovanadates, such as monomeric vanadate, which then oligomerizes to form decavanadate inside the cell. The formation of decavanadate has been demonstrated in yeast (*S. cerevisiae*) and thus makes this anion a desirable system to understand in greater detail (Willsky et al., 1984b, 1985).

**Figure 1:**
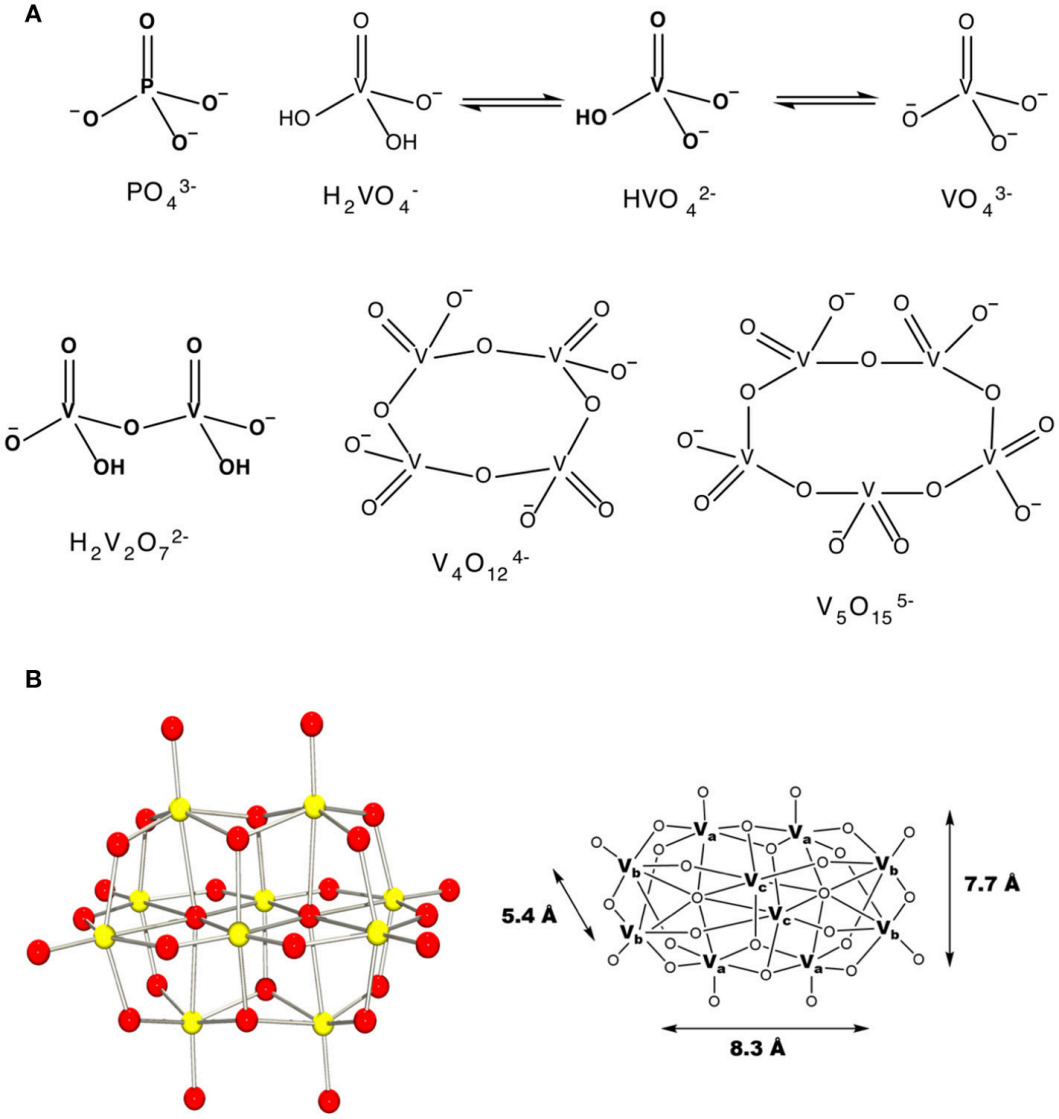
The schematic structures of **(A)** monomeric vanadate, monomeric and polymeric vanadate series **(B)** X-ray structure of V_10_ and the three different types of vanadium atoms in this complex ion with indicated dimensions (Evans, 1966). This figure was adapted with permission from Aureliano and Ohlin (2014), Crans et al. (2017).

Vanadium is a first-row transition metal ion and is in the group of transition metals that can form POMs (Baes, 1976; Chasteen, 1983; Vilas Boas and Costa Pessoa, 1987; Pope and Müller, 1991; Rehder, 1991; Crans et al., 2004, 2017). Vanadium is particularly prone to forming homopolyoxometalate ions as well (Baes, 1976; Aureliano and Crans, 2009). Indeed, pure crystalline metavanadate and orthovanadate upon dissolution will form several oxovanadate species containing vanadate monomer, V_1_, vanadate dimer, V_2_, vanadate tetramer, V_4_, vanadate pentamer, V_5_ and decavanadate, V_10_ (Pettersson et al., 1983, 1985; Crans et al., 1990), Figure 1. Some of these species have been characterized using X-ray crystallography and have been found to interconvert in aqueous solution (Evans, 1966; Crans et al., 1990). However, the specifics of the reactions and their conditions vary. For example, vanadate and oligomeric species containing 2, 4, and 5 vanadium atoms are colorless and rapidly convert at neutral pH, Figure 1A (Crans et al., 1990). In contrast, decavanadate will form rapidly at acidic pH, but is only kinetically stable at neutral pH, Figure 1B (Baes, 1976; Pope and Müller, 1991; Aureliano and Crans, 2009; Crans et al., 2017). Indeed, the kinetic studies have shown that decamer formation is a rapid process and much faster than the V_10_ decomposition in both neutral and basic solution (Clare et al., 1973a,b; Druskovich and Kepert, 1975; Comba and Helm, 1988; Kustin, 2015; Crans et al., 2017). The decomposition pathways investigated follow several different mechanisms and are dependent on the concentrations of H^+^ and OH^−^ and the other counter ions present in solution (Clare et al., 1973a,b; Druskovich and Kepert, 1975; Comba and Helm, 1988; Kustin, 2015). Information is needed describing how decavanadate interacts with membrane interfaces and cellular systems, including how decavanadates biological activities compare to monomeric vanadate. Specifically, an attractive alternative mode of action to simple decavanadate binding would be the direct delivery of a vanadium atom from decavanadate to a biomolecule resulting in the dissolution of the decavanadate cluster. The X-ray characterization of decavanadate shows that its dimensions are 5.4 Å × 7.7 Å × 8.3 Å, Figure 1 (Evans, 1966), a large size that cannot be accommodated by many biological transport channels. Therefore, any uptake of decavanadate is likely to be through endocytosis or a passive mechanism. An attractive alternative mode of uptake would involve dissolution of the cluster by direct delivery of vanadium atoms, for example, into a system such as a protein. However, such a mechanism is more difficult to investigate and will require more information regarding the potential interactions of the anion with ligands and interfaces. We have been addressing related questions for some time (Crans et al., 2017) and exploring the interactions of V_10_ with interfaces (Baruah et al., 2006; Crans et al., 2006, 2017; Samart et al., 2014; Sanchez-Lombardo et al., 2016). Indeed, more information is needed to be able to characterize the process of how the decavanadate converts to the vanadate monomer and smaller clusters, but such processes undoubtedly involve the molecular association of structures that form in solution. The work presented in this manuscript compares the effect of decavanadate and oxovanadates on two mycobacterial species. The results of this comparison are related to the questions of uptake of vanadate and will form the background information on which it will be possible to begin to address issues regarding potential delivery of V-atoms as well as uptake of decavanadate compared to monomeric and oligomeric vanadate.

Mycobacteria are particularly resistant to hydrophilic drug penetration, and thus studies with these organisms are of general interest. There have been several studies documenting the possibility that vanadium may affect growth of the bacteria, whether in a stimulatory of inhibitory manner; however, these studies did not consider the speciation of the studied compounds in growth media and in the biological context of the cells. For example, previous studies have been carried out investigating the effects of the simple salt vanadate on the cell growth of mycobacteria in the presence and absence of Tween-80 (Costello and Hedgecock, 1959). These studies investigated the possibility that vanadium may be stimulatory to growth because it would be able to replace Fe^2+^, Fe^3+^, or biologically active Mo-atoms (Turian, 1951). Dose response curves reported in cultures in the presence of added metavanadate gave complete growth inhibition observed at 5.0 μg/mL V-atom for *M. tb* (H37Rv), at 10 μg/mL V-atom for *Mycobacterium butyricum*, at 50 μg/mL V-atom for *Mycobacterium phlei*, but even at 200 μg/mL V-atom *M. smeg* was not completely inhibited (Costello and Hedgecock, 1959).

Additionally, studies have been carried out on vanadium coordination complexes and their effect on the growth of mycobacteria (Maiti and Ghosh, 1989; David et al., 2005; Correia et al., 2014; Gajera et al., 2015). The studies explore the possibility that a known drug, or drug derivative complexes binding to vanadium, may exert a greater synergetic activity (Correia et al., 2014; Gajera et al., 2015). For example, in a series of hydroxyquinoline (Correia et al., 2014), fluoroquinoline (Gajera et al., 2015) and acid hydrazides (Martins et al., 2015) vanadium(V) complex studies, the complexes were found with micromolar inhibitory potency against *M. tb*. In other systems the vanadium complex was found to have little if any effect on the growth of the bacterium (Maiti and Ghosh, 1989; Gajera et al., 2015). Additionally, a series of vanadium thiosemicarbazone and hydroquinoline complexes tested against *M. tb* were found to have micromolar inhibition constant against growth, but the ligand was more potent than the complex (Correia et al., 2014; Gajera et al., 2015). In these cases, complexation to the vanadium protected the bacterium. Combined these studies demonstrate that vanadium compounds may be inhibitory or protective, depending on the environment and ligands that may be complexed to it, and thus it is of interest to investigate the effects of simple vanadium complexes prior to formation of a coordination complex.

In this work, we determine the effects of decavanadate and the rapidly exchanging oxovanadates on the growth of two mycobacterial species. These studies directly compare the effects of the two vanadium salt species and in doing so, will allow researchers to address the issue of whether such oxometalate species exert different effects, possibly due to uptake or conversion of the salt under physiological conditions. In our studies with *M. smeg* and *M. tb*, it was discovered that decavanadate undergoes hydrolysis forming monomeric vanadate in the presence of the cells, suggesting some type of interaction with the bacterial cells or with the material excreted by the bacterial cells. Comparison of metavanadate and decavanadate demonstrates that these two oxovanadate species have very different effects on the growth of these mycobacterial species and depending on species, the potency varies.

## Materials and methods

### Materials

Sodium metavanadate (99.9% NaVO_3_) was purchased from Sigma-Aldrich, hydrochloric acid (36.5–38%, HCl) and citric acid (99% C_6_H_8_O_7_) were purchased from Fischer. Deuterium oxide (D_2_O, 99.9%) was purchased from Cambridge Isotope Laboratories, Inc. All reagents and other chemicals was purchased from Sigma-Aldrich. Vanadium complexes were prepared according to literature (Samart et al., 2014; Sanchez-Lombardo et al., 2016).

### Preparation of stock solutions for NMR spectroscopy evaluation

#### Vanadate

Colorless stock solutions of sodium metavanadate (NaVO_3_, 100 mM) were prepared in deuterium oxide (D_2_O). The suspension was heated to dissolve the solid, the solution was cooled to room temperature and then the pH was measured (~ pH 7–8) (Crans et al., 1990).

#### Decavanadate

An orange-red solution of sodium decavanadate (Na_6_V_10_O_28_, 100 mM) was prepared from NaVO_3_ (1.00 M, 1.22 g). Sodium metavanadate solutions were prepared directly by addition of solid NaVO_3_ to D_2_O and heating the solution to dissolve the solid. Once the solution was clear, the pH was adjusted to 4.5–5.8 using 6 M DCl (DCl was prepared from 12 M HCl by the addition of D_2_O) resulting in an orange colored solution. The V_10_ content in this solution was verified using ^51^V NMR spectroscopy and assuming the vanadium was all present as V(V) the concentration of each oxovanadate could be calculated from the integration of the spectra (Crans et al., 1990).

#### Citric acid and V-citrate complex solutions

Stock solutions of citric acid (100 mM, pH 2.33) were prepared in deuterium oxide (D_2_O). The vanadium-citrate complexes were prepared by adding equimolar amounts of citric acid and vanadium. The solution was diluted to form solutions with 75, 50, 10, and 5.0 mM vanadium(V).

#### Preparation of stock solution to cell culture studies

Stock solutions of vanadate and vanadium complexes were prepared in double distilled water (DI H_2_O), 7H9 media or in 100% DMSO depending on the solubility of these complexes. Specifically, metavanadate, orthovanadate or decavanadate were prepared in double distilled water. Isoniazid (INH) was dissolved in sterile distilled water and used as a positive control.

#### Nuclear magnetic resonance (NMR) measurements

All ^51^V NMR spectroscopy measurements were taken on a Bruker spectrometer at 78.9 MHz for ^51^V (400 MHz for ^1^H). The chemical shifts were obtained using external reference for ^51^V NMR of Na_3_VO_4_ (100 mM, pH 12.9, signals at −535 (V_1_) and −559 ppm (V_2_)). The samples were prepared fresh to form vanadium-citrate complexes in media and the composition was investigated as a function of time with experiments carried out at time points: 0, 1, 5, and 24 h.

Speciation of vanadium was calculated using the integration of the vanadium peak(s) within the ^51^V NMR spectra. The concentration of each species was determined using the known added concentration [assuming all vanadium is in the form of V(V)] (Crans et al., 1990), the integration of the vanadium peak(s) in the spectrum and by using the mole fractions for each signal, the concentration of each species could be calculated as shown in Table 1.

**Table 1:** Formation constants of Vanadate species in a 0.6M NaCl system.

**(p, q)**	**log β**	**Formula**	**Extended formula**
**BINARY SYSTEM (H**^+^**-H**_2_${\text{VO}}_{4}^{-}$**) REF (2)**			
−1, 1	−7.92	HV${\text{O}}_{4}^{2-}$	(H^+^)_−1_(H_2_V${\text{O}}_{4}^{2-}$)
−2, 2	−15.17	V_2_${\text{O}}_{7}^{4-}$	(H^+^)_−2_(H_2_V${\text{O}}_{4}^{2-}$)_2_
−1, 2	−5.25	HV_2_${\text{O}}_{7}^{3-}$	(H^+^)_−1_(H_2_V${\text{O}}_{4}^{2-}$)_2_
0, 2	2.77	H_2_V_2_${\text{O}}_{7}^{2-}$	(H_2_V${\text{O}}_{4}^{2-}$)_2_
−2, 4	−8.88	V_4_${\text{O}}_{13}^{6-}$	(H^+^)_−2_(H_2_V${\text{O}}_{4}^{2-}$)_4_
−1, 4	0.22	HV_4_${\text{O}}_{13}^{5-}$	(H^+^)_−1_(H_2_V${\text{O}}_{4}^{2-}$)_4_
0, 4	10.0	V_4_${\text{O}}_{12}^{4-}$	(H_2_V${\text{O}}_{4}^{2-}$)_4_
0, 5	12.4	V_5_${\text{O}}_{15}^{5-}$	(H_2_V${\text{O}}_{4}^{2-}$)_5_
4, 10	52.1	V_10_${\text{O}}_{28}^{6-}$	(H^+^)_4_(H_2_V${\text{O}}_{4}^{2-}$)_10_
5, 10	58.1	HV_10_${\text{O}}_{28}^{5-}$	(H^+^)_5_(H_2_V${\text{O}}_{4}^{2-}$)_10_
6, 10	61.9	H_2_V_10_${\text{O}}_{28}^{4-}$	(H^+^)_6_(H_2_V${\text{O}}_{4}^{2-}$)_10_
7, 10	63.5	H_3_V_10_${\text{O}}_{28}^{3-}$	(H^+^)_7_(H_2_V${\text{O}}_{4}^{2-}$)_10_
2, 1	6.96	V${\text{O}}_{2}^{+}$	(H^+^)_2_(H_2_V${\text{O}}_{4}^{2-}$)_10_
**(p, q, r)**	**log** β	**Formula**	**Extended formula**
**TERNARY SYSTEM (H**^+^**-H**_2_${\text{VO}}_{4}^{2-}$**C**_6_**H**_5_**${\text{O}}_{7}^{3-}$) REF (3**)			
1, 0, 1	5.217	Cit^2−^	(H^+^)(Cit^3−^)
2, 0, 1	9.298	Cit^−^	(H^+^)_2_(Cit^3−^)
3, 0, 1	12.067	Cit	(H^+^)_3_(Cit^3−^)
1, 2, 1	12.84	(H^+^)(H_2_V${\text{O}}_{4}^{2-}$)_2_(Cit^3−^)	
2, 2, 1	19.68	(H^+^)_2_(H_2_V${\text{O}}_{4}^{2-}$)_2_(Cit^3−^)	
3, 2, 1	24.12	(H^+^)_3_(H_2_V${\text{O}}_{4}^{2-}$)_2_(Cit^3−^)	
3, 1, 1	18.35	(H^+^)_3_(H_2_V${\text{O}}_{4}^{2-}$)(Cit^3−^)	
2, 1, 1	14.1	(H^+^)_2_(H_2_V${\text{O}}_{4}^{2-}$)(Cit^3−^)	
4, 2, 2	31.3	(H^+^)_4_(H_2_V${\text{O}}_{4}^{2-}$)_2_(Cit^3−^)_2_	
5, 2, 2	35.3	(H^+^)_5_(H_2_V${\text{O}}_{4}^{2-}$)_2_(Cit^3−^)_2_	
6, 2, 2	39.2	(H^+^)_6_(H_2_V${\text{O}}_{4}^{2-}$)_2_(Cit^3−^)_2_	
**(p, q, r)**	**log** β	**Formula**	**Extended formula**
**TERNARY SYSTEM (H**^+^**-H**_2_${\text{VO}}_{4}^{2-}$**H**_2_${\text{PO}}_{4}^{-}$**) REF (4)**			
−2, 0, 1	−17.650	P${\text{O}}_{4}^{3-}$	[(H^+^)_−2_(H_2_P${\text{O}}_{4}^{-}$)]^3−^
−1, 0, 1	−6.418	HP${\text{O}}_{4}^{2-}$	[(H^+^)_−1_(H_2_P${\text{O}}_{4}^{-}$)]^2−^
1, 0, 1	1.772	H_3_PO_4_	[(H^+^)(H_2_P${\text{O}}_{4}^{-}$)]
9, 14, 1	90.7	H_3_PV_14_${\text{O}}_{42}^{6-}$	[(H^+^)_9_ (H_2_V${\text{O}}_{4}^{2-}$)_14_ (H_2_P${\text{O}}_{4}^{-}$)]^6−^
10, 14, 1	94.84	H_4_PV_14_${\text{O}}_{42}^{5-}$	[(H^+^)_10_ (H_2_V${\text{O}}_{4}^{2-}$)_14_ (H_2_P${\text{O}}_{4}^{-}$)]^5−^
11, 14, 1	96,41	H_5_PV_14_${\text{O}}_{42}^{4-}$	[(H^+^)_11_ (H_2_V${\text{O}}_{4}^{2-}$)_14_ (H_2_P${\text{O}}_{4}^{-}$)]^4−^

#### Speciation analysis

The interpretation of the ^51^V NMR spectral data was supplemented by speciation calculations based on constants measured previously (Pettersson et al., 1983; Ehde et al., 1989; Selling et al., 1994). The species distribution diagrams were calculated by using HySS 2009 software (Alderighi et al., 1999; Carsella et al., 2017) and known speciation constants of the system at hand (Pettersson et al., 1983; Ehde et al., 1989; Selling et al., 1994). The citrate and the phosphate concentrations found in the Middlebrook 7H9 broth medium supplemented with 5% BSA, 2% dextrose, 5% catalase (ADC) enrichment, glycerol (0.2%, v/v), and Tween 80 (0.05%, v/v) were 0.48 and 24 mM, respectively (Bbl^*tm*^, 2018). The vanadium concentrations that were investigated were concentrations of 5, 3.3, and 10 mM. The speciation diagrams were constructed using the following equilibrium reactions for the binary H^+^-H_2_V${\text{O}}_{4}^{2-}$ system [see equation (1)] (Pettersson et al., 1983), and two ternary system, H^+^-H_2_V${\text{O}}_{4}^{2-}-$C_6_H_5_${\text{O}}_{7}^{3-}$ and H^+^-H_2_V${\text{O}}_{4}^{2-}-$H_2_P${\text{O}}_{4}^{-}$ [see equation (2) (Ehde et al., 1989) and (3) (Selling et al., 1994)]. The alternate formulas were provided in the format of the equations provided for comparison.

(1) $$p{\text{H}}^{+}+q{\text{H}}_{2}{\text{VO}}_{4}^{-}⇄{\left({\text{H}}^{+}\right)}_{p}{\left({\text{H}}_{2}{\text{VO}}_{4}^{2-}\right)}_{q}$$

(Pettersson et al., 1983)

(2) $$p{\text{H}}^{+}+q{\text{H}}_{2}{\text{VO}}_{4}^{2-}+r{\text{C}}_{6}{\text{H}}_{5}{\text{o}}_{7}^{3-}⇄{\left({\text{H}}^{+}\right)}_{p}{\left({\text{H}}_{2}{\text{VO}}_{4}^{2-}\right)}_{q}{\left({\text{C}}_{6}{\text{H}}_{5}{\text{o}}_{7}^{3-}\right)}_{r}$$

(Ehde et al., 1989)

(3) $$p{\text{H}}^{+}+q{\text{H}}_{2}{\text{VO}}_{4}^{2-}+r{\text{H}}_{2}{\text{PO}}_{4}^{-}⇄{\left[{\left({\text{H}}^{+}\right)}_{p}{\left({\text{H}}_{2}{\text{VO}}_{4}^{2-}\right)}_{q}{\left({\text{H}}_{2}{\text{PO}}_{4}^{-}\right)}_{r}\right]}^{\text{p}-\text{q}-\text{r}}$$

(Selling et al., 1994)

As described previously, the equations above describe the nature of the complexes that form. For example, HV${\text{O}}_{4}^{2-}$ is described as H_2_V${\text{O}}_{4}^{-}$ minus H^+^ and thus the species is described as (−1,1) where the p being −1 (Pettersson et al., 1983; Ehde et al., 1989).

#### Bacterial strains and culture conditions

*Mycobacterium smegmatis* mc^2^155 was grown in 7H9 Middlebrook Medium supplemented with 0.2% (v/v) glycerol, 10% ADC, 0.05% Tween-80, and incubated at 37°C with shaking for 24 h. The growth of bacteria was monitored to mid-logarithmic growth phase using a spectrophotometer at 600 nm to an optical density of 0.6 (OD_600nm_) (Bbl^*tm*^, 2018). Metavanadate (40 mM, pH 8.6) and decavanadate [100 mM V_10_ (1.0 M V-atoms), pH 3.8] stock solutions and the media with bacteria were added to a 96 well plate in a 3-fold dilution experiment monitored at times 0, 1, 5, and 24 h. The pH of these samples ranged from 5.5 to 6.3 before and after treatment with V-compounds and growth; see spectra for in supplemental for details (Upadhyay et al., 2015).

Culture supernatant after cell growth generated for ^51^V NMR analysis was prepared by running separate growth experiments for the *M. smeg* mc^2^ 155 experiments with a total volume of 5 mL. At each time point, a 1 mL aliquot was removed for NMR analysis. ^51^V NMR spectra were run at the time points 0, 1, 5, and 24 h without lock.

*M. tb* mc^2^ 6230 is a nonpathogenic deletion mutant (ΔRD1 ΔpanCD) strain H37Rv that can safely be used in a Biosafety Level 2 conditions (Sambandamurthy et al., 2006). The bacteria were grown in 7H9 Middlebrook medium with the addition of D-pantothenate (24 mg/L) at 37°C to an optical density at 600 nm (OD_600nm_) of 0.6–0.8. The cultures were diluted and treated with the appropriate metavanadate and decavanadate stock solutions in a 96 well plate in a 3-fold dilution experiment, and incubated at 37°C for the duration of the experiment (5–7 days). The pH values of these samples ranged from 5.6 to 6.8 after growth for 5 days; see spectra for details.

^51^V NMR spectra were acquired on culture supernatant without lock (no D_2_O in sample) at the beginning and end of the bacterial growth experiment (0 and 5 days) for *M. tb* mc^2^ 6230 growth samples.

## Results

Investigating the effects of both vanadate and decavanadate on mycobacterial growth requires information on the speciation of vanadium under the conditions of the growth assay studies. *M. tb* and *M. smeg* were grown in supplemented Middlebrook 7H9 medium. Some of the components of the medium may form vanadium complexes. The major candidates for complex formation based on literature formation constants are phosphate (Gresser et al., 1986), citrate (Pettersson et al., 1985; Levina et al., 2017a), and amino acids (Crans, 2000; Rehder et al., 2002; Esbak et al., 2009). In the following, we investigated whether these complexes formed in the media, Figure 1.

### Reaction of vanadate and decavanadate with components in growth media

**Figure 2:**
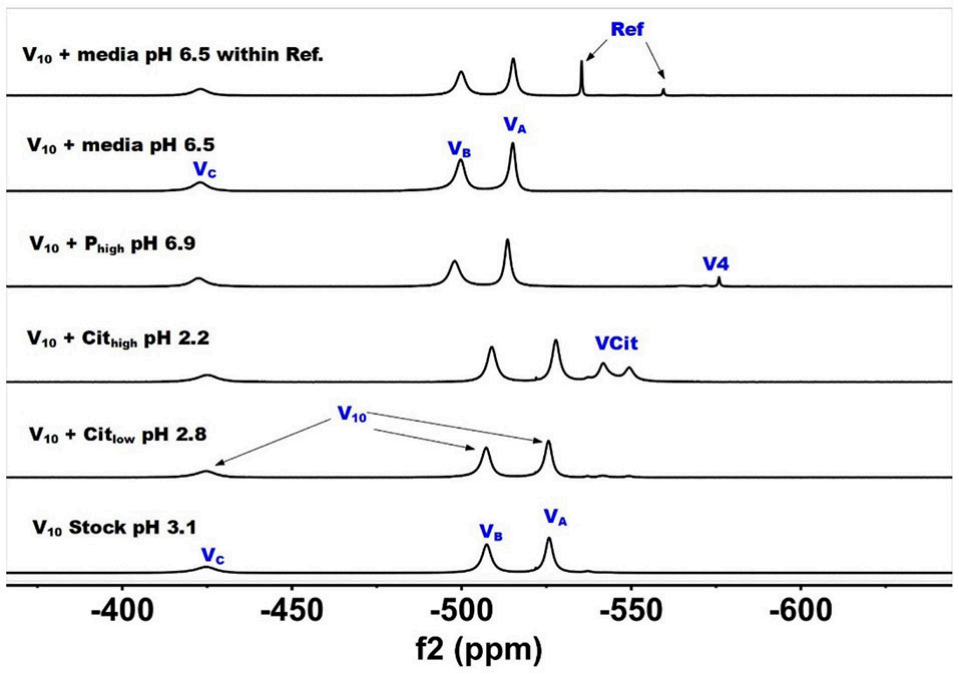
^51^V NMR (78.9 MHz) spectra are shown of solution of decavanadate (10 mM V_10_, 100 mM V-atoms). The samples are from the bottom up diluted V_10_ stock solution (100 mM V-atom) at pH 3.1; 10 mM V_10_ in the presence of 0.48 mM and 0.97 mM citrate at pH 2.8 and 2.2, respectively; 10 mM V_10_ in the presence of 24 mM P_i_ at pH 6.9; and finally 10 mM V_10_ in the presence of Middlebrook 7H9 broth medium supplemented with 10% ADC enrichment (5% BSA, 2% dextrose, 5% catalase), glycerol (0.2%, v/v) and Tween 80 (0.05%, v/v) recorded both in the absence and the presence of a capillary reference of 100 mM Na_3_VO_4_.

**Figure 3:**
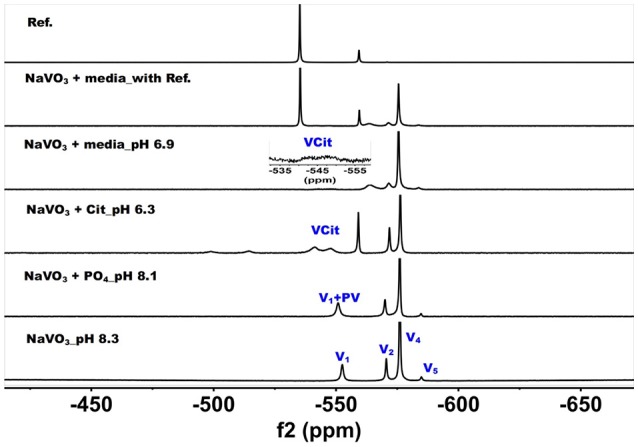
^51^V NMR (78.9 MHz) spectra are shown of solution of colorless oxovanadate (40 mM V_1_, 40 mM V-atoms). The samples are from the bottom up diluted V_1_ stock solution (40 mM V-atom) at pH 8.3; 10 mM V_10_ in the presence of 24 mM P_i_ at pH 8.1; 10 mM V_10_ in the presence of 0.48 mM citrate at pH 6.3, and finally 10 mM V_10_ in the presence of Middlebrook 7H9 broth medium supplemented with 10% ADC enrichment (5% BSA, 2% dextrose, 5% catalase), glycerol (0.2%, v/v) and Tween 80 (0.05%, v/v) recorded both in the absence and the presence of a capillary reference of 100 mM Na_3_VO_4_. The spectrum labeled Reference is of the capillary reference alone (top spectrum). The key to the signals: V-oligomers, V_1_ monomer; V_2_, dimer; V_4_, tetramer; V_5_, pentamer; VCit, V-citrate complex; PV, vanadate-phosphate complex.

**Figure 4:**
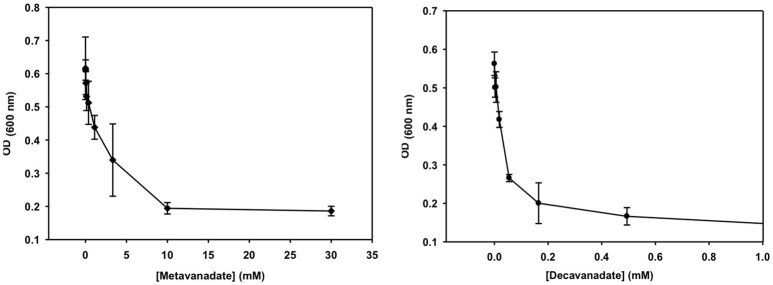
The growth curve is shown for treatment with vanadate (left, prepared from a 40 mM colorless metavanadate solution) and decavanadate [right, prepared from a 100 mM orange decavanadate solution (1.0 M V-atoms) on *M. tb*].

^51^V NMR studies were carried out with vanadate and decavanadate solutions and Middlebrook 7H9 broth medium supplemented with 10% ADC enrichment (5% BSA, 2% dextrose, 5% catalase), glycerol (0.2%, v/v) and Tween 80 (0.05%, v/v). The mycobacteria grow well at pH 6.8, and decavanadate is known to form between pH 3 to 6.5 (Pope and Müller, 1991; Crans et al., 2004; Baruah et al., 2006; Aureliano and Crans, 2009). V_10_ is formed at low pH values and the V_10_ hydrolyzes at neutral pH. When V_10_ is added to the growth media the pH was increased from pH 7.6 to 6.5/7 by the buffering capacity of the growth media and there is a potential for hydrolysis of V_10_. Importantly, little hydrolysis is observed in growth media in the absence of mycobacteria. NMR spectra were recorded at a range of pH values because the reactions of vanadate are very sensitive to pH (see Supplemenal Material); however, the spectra shown in Figures 2 and 3 are those of direct relevance to observation and assignment of the species that form in the growth media (see below Figure 4). Importantly, all the spectra are referenced against a reference sample (labeled Ref. in Figure 3, containing 100 mM Na_3_VO_4_).

In Figure 2, we show the spectra of decavanadate under various conditions. First, we show the spectrum of the stock solution at pH 3.1 with the three characteristic signals for the three different V-atoms in the V_10_ molecule. Next, we show two spectra with added citrate at two concentrations at pH 2.8 and 2.2 where the decavanadate will form VCit complex (two signals at −548 and −552 ppm). The next three spectra at higher pH values are shown first in the presence of phosphate (P_i_), demonstrating that the signals for V_10_ shifts as the pH increases from 3 to 6.9. As shown in the final two spectra in Figure 2, V_10_ in the presence of media that contains both citrate and P_i_ did not form any other V-products when all V-atoms are in the form of V_10_. The spectrum of V_10_ in media is shown both in the presence and absence of a capillary tube filled with Na_3_VO_4_ and used to reference the spectra (Figure 2). All the NMR spectra were referenced in this manner and the other NMR spectra are provided in the Supplemental Material. In summary, these spectra shown that when vanadium is present in the form of V_10_, other vanadium species do not form in the cell media.

The spectra shown in Figure 2 demonstrated that the V_10_ is stable in the growth media, which is consistent with studies in yeast media reported previously (Willsky et al., 1984a, 1985). The data shown in Figure 2 demonstrated that at pH 2, the V_10_ forms complexes with citrate but as the pH increased to near neutral pH, V_10_ did not form such products with growth media components (Ehde et al., 1989; Zhou et al., 1999; Crans, 2000; Kaliva et al., 2002, 2003; Chen et al., 2007, 2014; Lodyga-Chruscinska et al., 2008). In contrast, when V_10_ is converted to the oxovanadates or V_1_ is the starting form at vanadium, the VCit and PV complexes are formed (this is generally observed in solutions at neutral or more basic pH values, Figure 3). However, if the pH of the growth media decreases (that is the acidity increase and pH value decrease), V_10_ hydrolyzes and forms oxometalates that are able to react with media components (see Figures 2, 3).

The spectra of solutions from metavanadate (NaVO_3_) containing vanadate monomer and other oxovanadates prepared in aqueous solution and in media are shown in Figure 3. Since vanadate solutions contain rapidly converting species (Crans et al., 1990) and the composition depends on pH and concentration, the species present is dependent on the solution composition. The first spectrum shows a solution containing the interconverting oxovanadates, V_1_, V_2_, V_4_, and V_5_. In the presence of P_i_ and at slightly lower pH value, the V_1_ signal shifted and the linewidth increased, which is indicative of the formation of a vanadate-phosphate complex (abbreviated PV on the spectra in Figure 2) (Gresser et al., 1986; Andersson et al., 2005). Although there are no X-ray structures for these species, the Gresser-Tracey team proposed that there is both a vanadium species that is four or five-coordinate and octahedral based on the chemistry shifts). The formation of the PV species (Gresser et al., 1986; Andersson et al., 2005) is rapid on the NMR time scale, and thus results in shifting of the vanadate monomer (V_1_) signal in place of observing two separate signals one for V_1_ and one for PV. As the pH decreased to 6.3, V_1_ protonates and the monomer signal shifts to about−560 ppm. At these higher pH values, the VCit complex is found to form in solutions containing both V_1_ and citrate. Many different VCit complexes are known, however, the broad doublet signal observe for the VCit could be several structures as described in the literature (Ehde et al., 1989; Zhou et al., 1999; Crans, 2000; Kaliva et al., 2002, 2003; Chen et al., 2007, 2014; Lodyga-Chruscinska et al., 2008). The final spectra show the addition of the vanadate stock solution to media, which at pH 6.9 results in a solution that contains a signal of V_1_ and PV, V_2_, and V_4_ and a trace of V_5_. The amount of VCit formed is low and it is difficult to see the signal from in the normal growth media spectrum, so we used an increased amplification to show the VCit signal and part of such spectrum is shown in the spectral insert.

In summary, if the growth of the mycobacteria is conducted near pH 6.8, the speciation of vanadium (V) will readily reach thermodynamic control. Solutions prepared from NaVO_3_ will contain little to no V_10_ and solutions containing V_10_ will be relatively stable. Therefore, growth studies will be carried out with either V_1_ or V_10_ species, and it will be possible to determine if there is a difference in the growth effects on mycobacteria by these two species. That is the growth effects of the V_1_ with the oligomeric oxovanadates and V_10_ are measured in media with known vanadium speciation and under conditions where it is possible to observe the effect of each species.

### Growth inhibition experiments of vanadate monomer and decavanadate

Growth inhibition experiments were designed to measure the effects of V_10_ and V_1_ each on *M. tb* mc^2^ 6230 and *M. smeg* mc^2^ 155. The measurements monitor growth using absorbance at 600 nm and the concentration of the vanadium compound was changing with 3-fold dilution experiments. In this manner, the growth of the bacteria was measured over a 2,000-fold range of concentrations of vanadium compound. The results with the *M. tb* are shown in Figure 4, and the EC_50_ (concentration of compound where growth is reduced by half) value calculated for the V_1_ experiment was 2.0 mM (2.0 mM V-atoms) and for the V_10_ experiment 29 μM (0.29 mM V-atoms, Table 2).

**Table 2:** The EC_50_ values for V_1_ and V_10_ treated *Mycobacterium tuberculosis* and *smegmatis*.

	**EC_50_ (*M. tb*) (mM)**	**Stand. error**	**EC_50_ (*M. tb*) (mM V-atoms)**	**Stand. error**	**EC_50_ (*M. smeg*) (mM)**	**Stand. error**	**EC_50_ (*M. smeg*) (mM V-atoms)**	**Stand. error**
V_1_	2.0	0.43	2.0	0.43	0.19	0.071	0.19	0.071
V_10_	0.029	0.005	0.29	0.05	0.0037	0.0004	0.037	0.004

These results show that V_10_ is a more potent inhibitor than V_1_ by a factor of 100 in terms of concentration and a factor of 10 in terms of concentration of V-atoms. This result demonstrates that the large V_10_ anion is more inhibitory than the V_1_ atom even when counting for the fact that there are 10 V-atoms in each V_10_ species. As discussed below, ^51^V NMR spectroscopy was used to examine the speciation in the solutions and verify that the V_10_ solution indeed contained V_10_ (see below for details).

The growth effects were also measured for *M. smeg* mc^2^ 155 for both V_10_ and V_1_ (data not shown). Similarly, the EC_50_ values were calculated for the V_1_ experiment to be 190 μM (0.190 mM V-atoms) and for the V_10_ experiment to be 3.7 μM (0.037 mM V-atoms). These results show that the V_10_ is a more potent inhibitor than V_1_, by a factor of 100 in terms of concentration and a factor of 10 in terms of concentration of V-atoms as we also observed for *M. tb*.

These results present evidence that the large compact V_10_-anion is a stronger inhibitor than V_1_ of cell growth of the two mycobacterial strains. The more potent inhibition by V_10_ compared to V_1_ shows that the V_10_ is an inhibitor, and that it is not V_1_ formed from hydrolyzed V_10_. However, it should be mentioned that it is solutions of V_10_ that are causing the inhibitory growth effect, and we cannot rule out the possibility that the effect is caused by several species including the hydrolysis products, or some mechanism in which V_10_ delivers the V-atom to the cells. Regardless, these studies demonstrate the effects of simple oxovanadates and the large decavanadate. The fact that V_10_ has a greater effect than V_1_ (or 10 V_1_ molecules) supports the interpretation that the growth effects observed are not due to V_1_ formed from hydrolyzed V_10_. To support this interpretation, we acquired the ^51^V NMR spectra of the growth media solutions at various times during the growth experiment.

### Speciation studies in media of mycobacteria treated with vanadate and decavanadate

#### ^51^V NMR spectra of media that have grown mycobacteria and have been treated with metavanadate

**Figure 5:**
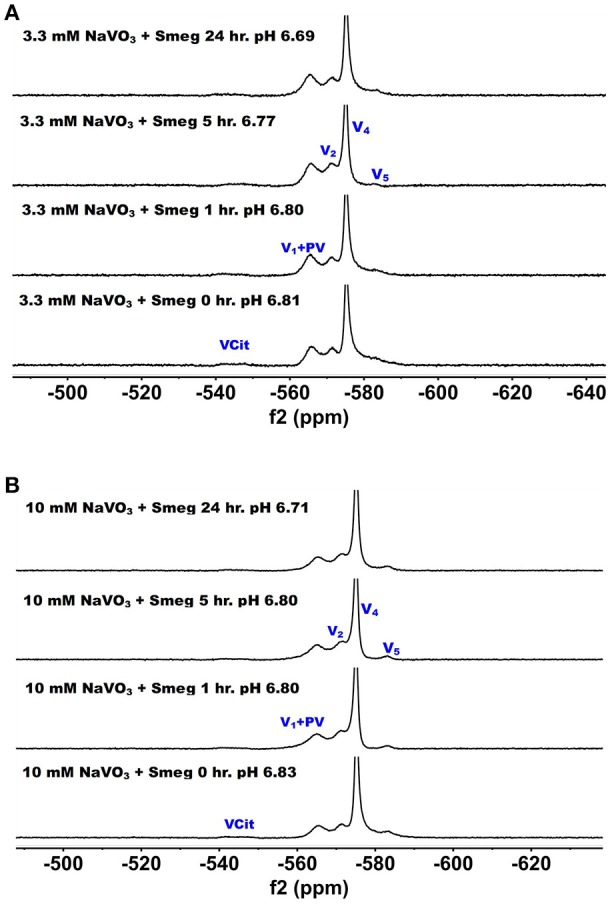
The ^51^V NMR spectra of media in which *M. smeg* had grown at 0, 1, 5, and 24 h time points. Data for two concentrations are shown in **(A)** 3.3 mM V_1_ treatment (3.3 mM V-atoms) and **(B)** 10 mM V_1_ treatment (10 mM V-atoms). See Figure 3 caption for key to labeling.

^51^V NMR spectra were recorded at several concentration and time-points in the growth media in which *M. smeg* had grown because as mentioned above the bacteria were subjected to a 2000-fold concentration range. In Figure 5, we show the ^51^V NMR spectra recorded at 3.3 mM and 10 mM oxometalates, respectively. Both these series show the presence of the different oxometalates (V_1_, V_2_ and V_4_). In addition, neither series show any evidence for change as the experiment proceeded. This is somewhat surprising, because experiments with other cellular systems, such as yeast, fungi and red blood cells, all showed significant signs of signal reduction (Willsky et al., 1984b, 1985; Crans et al., 2002; Delgado et al., 2005; Jakusch et al., 2014). Since the V_1_ or oxovanadate is not a strong inhibitor of growth, it is possible that the simple vanadate system is not getting into the *M. smeg* cells very effectively.

#### ^51^V NMR spectra of media in which mycobacteria have been treated with decavanadate

**Figure 6:**
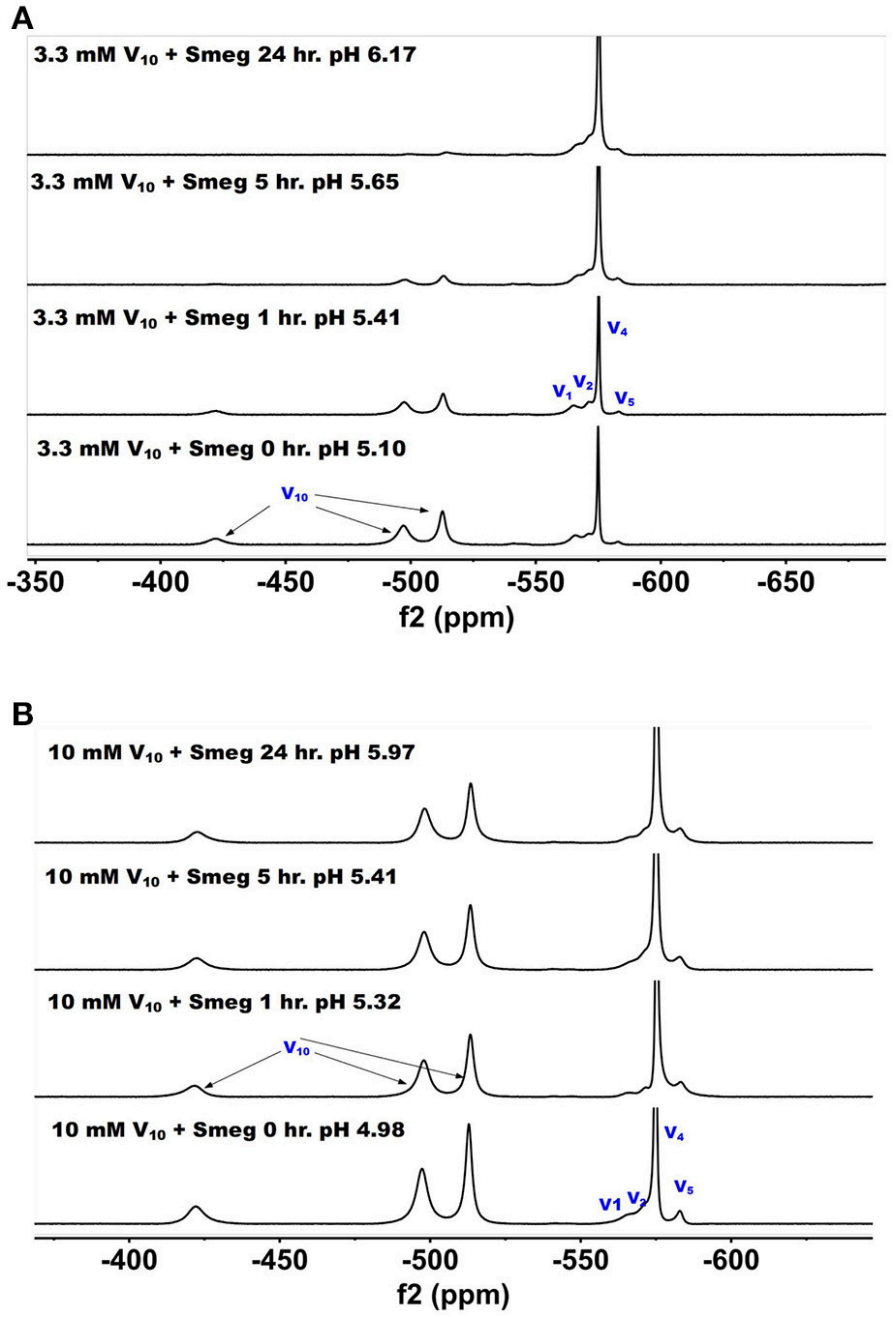
The ^51^V NMR spectra of media in which *M. smeg* had grown at 0, 1, 5, and 24 hr time points. Data for two concentrations are shown in **(A)** 3.3 mM V_10_ treatment (33 mM V-atoms) and **(B)** 10 mM V_10_ treatment (100 mM V-atoms).

^51^V NMR spectra were recorded at several concentrations and time-points in the growth media in which *M. smeg* had grown. In Figure 6, we show the ^51^V NMR spectra recorded at 3.3 and 10 mM, respectively. Both series show the presence of V_10_. Although the stock solutions and growth media without bacterial cells contains 100% V_10_ (Figure 2), the addition of growth media containing cells immediately caused some decomposition of V_10_ (Figure 6). NMR spectra were recorded multiple times of the 3.3 mM V and resulted in approximately a 1:1 ratio of V_10_ to oxovanadates as determined by integration of the spectra. As shown in Figure 6, after 1 h the composition of the sample was very similar to the content at the beginning of treatment, whereas at the 5 h time point the V_10_ had decreased significantly and at 24 h very little was left. This demonstrates that the V_10_ is not stable in the media in the presence of the *M. smeg* cells at 33 mM total V-atom concentration even though the pH is between 5.8 and 6.8 which is a pH range where there should not have been any hydrolysis of V_10_ according to the known solution chemistry of V_10_. However, V_10_ treatment may cause signal reduction or the V(V) may interact with large structural components in the mycobacterial cells (or their excretion products) and the ^51^V NMR signal has broadened beyond detection. Precedent for these possibilities has been reported previously (Willsky et al., 1984b, 1985; Crans et al., 2002). In the series of spectra at 10 mM V_10_ in the media, there is less evidence for V_10_ removal is apparent at the different time points. However, when we integrated the signals a slow decrease in the V_10_ signals compared to the oxovanadate signals was clear. The ratio at time zero was 1:0.71, at time 1 h it was 1:1.08; at time 5 h it was 1:1.18 and at 24 h it was 1:1.24. Thus, providing evidence for a slow decrease in V_10_ content as the experiment progressed. This data is consistent with the possibility that there is some reduction of the vanadium from the POM resulting in hydrolysis and formation of the oxovanadates.

To further investigate whether there was any reduction taking place upon vanadium treatment, we also measured the color change in these samples, Table 3. More data and the pH measurements on all the samples shown are provided in the Supplemental Material, and the pH values are all in the range from 6.1 to 7.4. Vanadium(V) on reduction is known to change from colorless (V_1_ oxovanadates) or yellow/orange (V_10_) to a green color (Crans et al., 2004, 2010, 2013; Pessoa and Tomaz, 2010; Jakusch et al., 2014). The 7H9 media alone did not cause any reduction as evidenced by the media sample maintained the color prior to treatment, Table 3. All the samples containing media and *M. smegmatis* treated with V_10_ developed a greenish tint as the treatment progressed beyond 5 h time points, whereas the samples treated with V_1_ remained colorless, see Table 3. This is consistent with reduction of some of the V(V) in the V_10_ salt. Interesting, similar reduction was not observed for the samples treated with the V_1_ solutions. These observations are consistent with the selective reduction of the V(V) and that the reduction was more prevalent after longer treatments, higher concentrations of vanadium and V_10_ compared to V_1_. Since the reduction only took place in the presence of V_10_, this anion induced a response that was not induced by V_1_. Furthermore, the supernatant of a batch of grown *M. smeg*. bacteria and centrifuging down the bacteria and removing them was collected to investigate if the bacteria excrete a component responsible for the observed reduction. The V_10_ and V_1_ samples were added to this supernatant and the data are shown in column 4 in Table 3. This supernatant also generate the color change from orange to green upon incubation. This experiment showed that the reduction is something that was induced by a material excreted from the *M. smeg*. cells during growth. This suggest that the reduction is likely to come from the excretion of siderophores (Hider and Kong, 2010) or proteins such as the reported protein tyrosine phosphatases from mycobacteria (Beresford et al., 2009; Dutta et al., 2016). Since we also carried out such studies of supernatant that had been heated (see Supplemental Material), such a product is either a very heat stable protein or a siderophore-type of material.

**Table 3:**
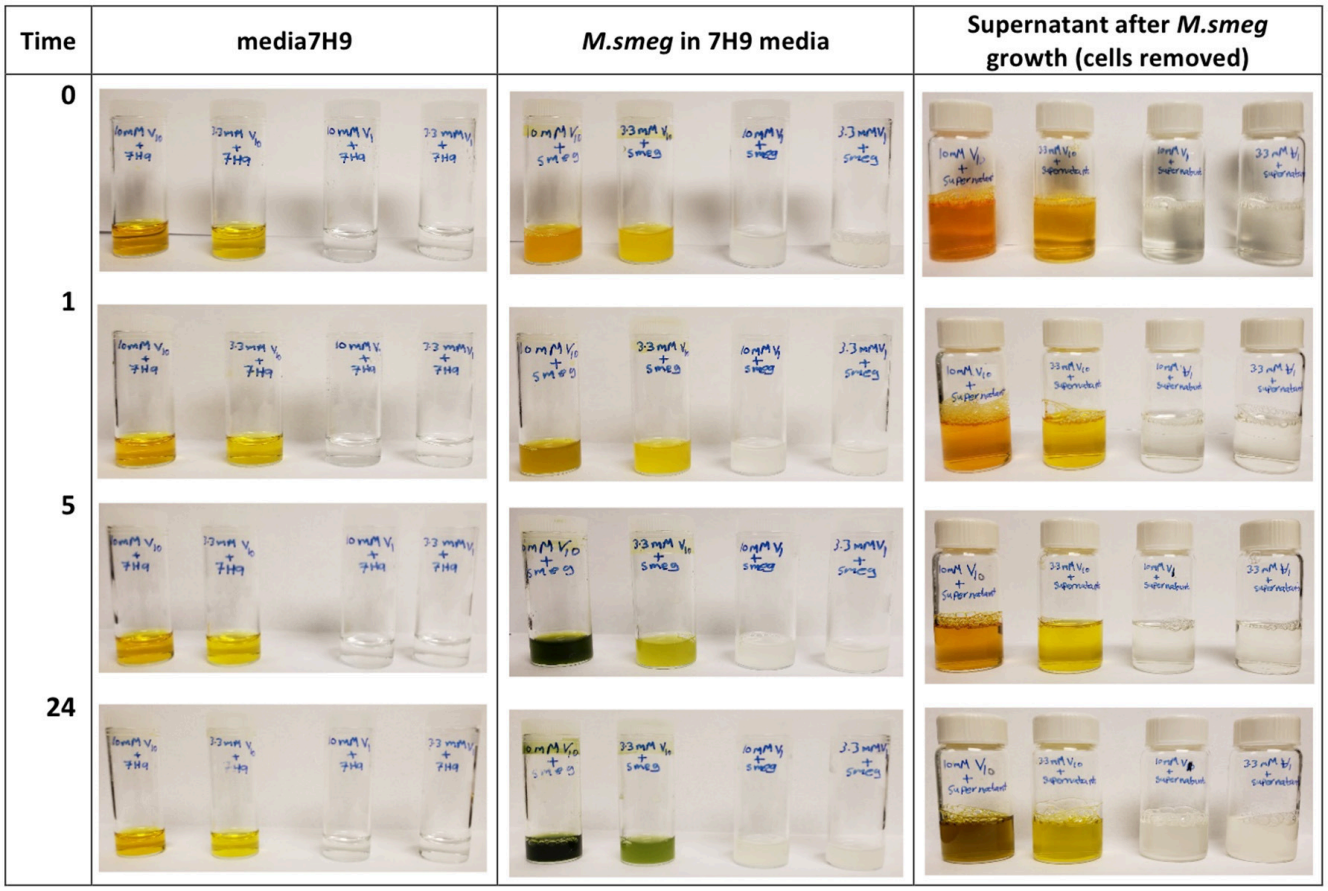
Samples treated with V_10_ and V_1_ in 7H9 media, *M. smeg* cultures and supernatant after *M. smeg*.

*Growth (columns 2, 3, and 4). The yellow or orange V_10_ is shown from the left with 10 and 3.3 mM samples, and the colorless V_1_ samples from the left 10 and 3.3 mM in each column. When reduction took place the green color is evident [vanadium (IV)]. See Supplemental Material for the pH values of each of these samples*.

Combined these studies show that there is selectivity in the effects of interactions of vanadium with mycobacteria, and that for these systems the most effective form is not the V_1_, which is a potent phosphatase inhibitor and a common active form of vanadium. These studies show that the V_10_, a POM is more potent inhibitor of growth of mycobacteria than the well-known phosphatase inhibitor, V_1_. The studies also demonstrate that there is some redox chemistry involved in this process and that the mycobacteria excrete a component that interact with V_10_ and cause the redox chemistry and hydrolysis of the V_10_. Eventually the V_1_ samples would also show the color change consistent with reduction, but this required growth periods beyond several weeks and this process may be different than the reduction, of V_10_.

### Modeling speciation vanadium species distribution in mycobacteria media added metavanadate

To quantify the V-species that are present in the growth assay media, we carried out speciation analyses modeling experimentally the distribution of vanadium species in the media. This analysis is based on analyzing solutions containing V-species that are governed by thermodynamics and can be calculated the HySS program (Alderighi et al., 1999). This model can analyze the experimental conditions found in solution prepared by the addition of metavanadate and exposed to the conditions of the cell growth assay. We used the reactions reported previously with regard to the exchange of labile oxovanadates (Pettersson et al., 1983, 1985; Gresser et al., 1986; Ehde et al., 1989; Crans et al., 1990, 2000; Crans, 2000, 2005; Andersson et al., 2005; Baruah et al., 2006; Aureliano and Crans, 2009), the formation of the Vcit complex (Ehde et al., 1989; Crans, 2000), the formation of the PV complex (Gresser et al., 1986; Andersson et al., 2005), and the thermodynamics relating to the decavanadate deprotonation reactions (Crans, 2005; Baruah et al., 2006; Aureliano and Crans, 2009). The known speciation parameters used for vanadate at various pH values were measured in the presence of 0.6 M NaCl to keep the salt concentration constant for all the components in the system. As shown in Figure 5, the species composition for the conditions of three different concentrations of vanadium (0.0050, 3.3, and 10 mM) are illustrated. As discussed above, the vanadium samples prepared with decavanadate will not contain this speciation distribution because all V(V) was converted to V_10_. The addition of this solution to the media will only slowly hydrolyze the V_10_ species to the equilibrium oxovanadate mixture if above or near pH 7.

The speciation analysis of the conditions observed in the media with freely exchangeable oxovanadate shown in Figure 3 demonstrate that an observable amount of VCit complex forms. Depending on the concentration of the vanadium, the contribution of VCit is larger percentage-wise at the lower concentrations of vanadate; for example, at 0.005 and 1.0 mM V-atoms at neutral pH the amount is 75–80% (in terms of mole fraction) of VCit complex. In contrast, at 10 mM V-atoms at neutral pH the amount is ~10%. However, if the amounts are calculated, this would correspond to 0.0004, and 1.0 mM VCit complex in the media. These observations are consistent with the very strong formation constant for VCit. The PV complex, however, is much less stable, and even though there is much more phosphate in the assay, the PV complex is only observed in a significant concentration at high V and P_i_ concentrations (5 and 24 mM, respective). Although the PV complex is only observed in high concentrations at mM V-treatments the shifting of the V_1_ signal attests to the presence of the PV adduct in the assays with *M. tb* and *M. smeg*.

**Figure 7:**
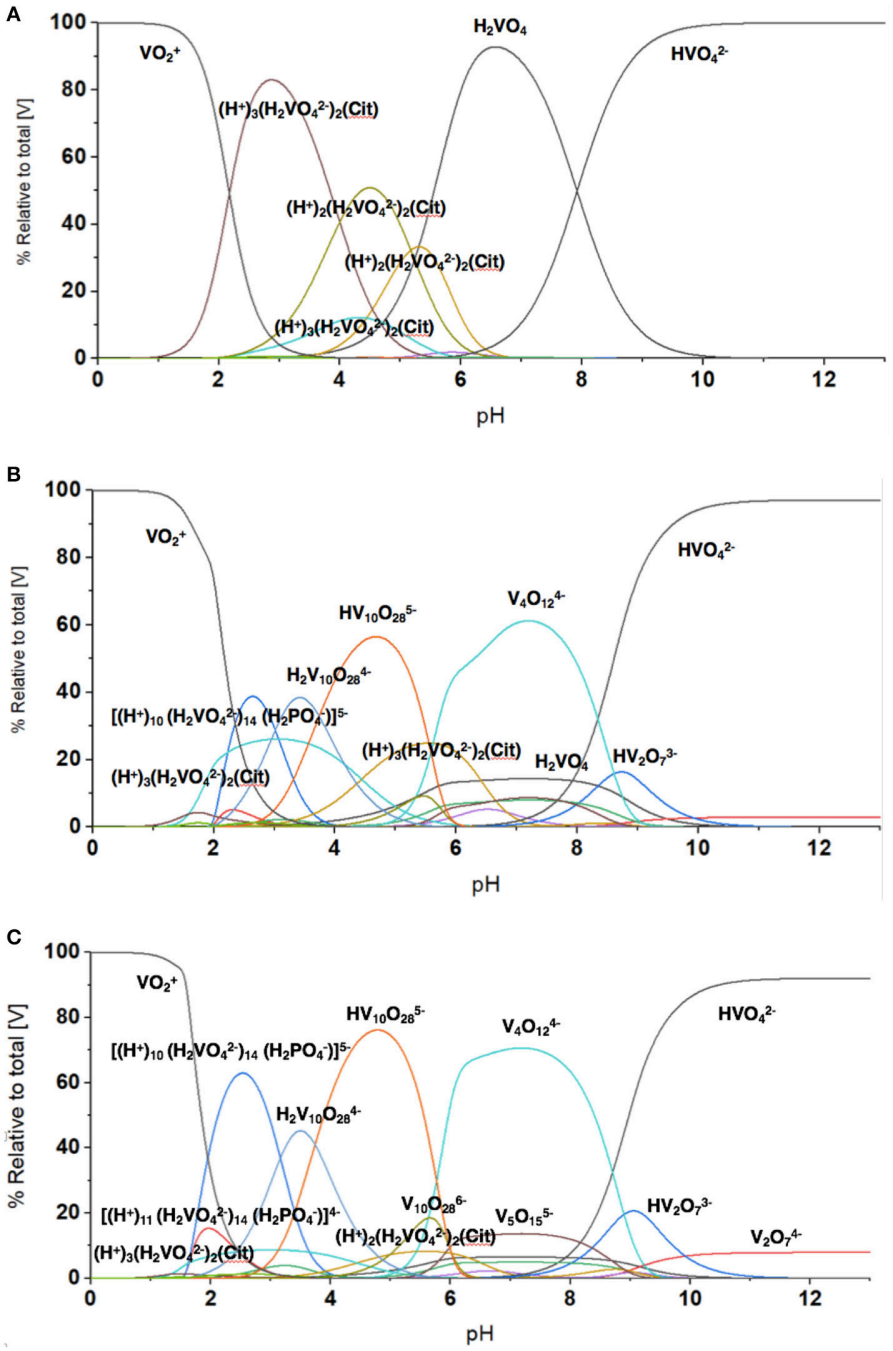
The evaluation of the speciation at different vanadium concentration using HySS Program (vs. 2009) (Alderighi et al., 1999). The speciation diagram shown was calculated at several vanadium (V-atom) concentrations in the presence of 0.48 mM citrate and 24 mM phosphate found in the growth media and **(A)** 5.0 μM vanadate, **(B)** 3.3 mM vanadate, and **(C)** 10 mM vanadate. Note, the concentrations of different species regardless of nuclearity are here shown in terms of V-atoms.

The results shown in Figure 7 are in general agreement with the experimental data obtained in Figures 2, 3, 5, 6 with regard to formation of the VCit and PV complexes. Figure 7 shows that there are four different VCit complexes formed at low vanadium concentration, but at higher concentration and in the presence of phosphate two major VCit complexes formed. There are several assumptions on which these estimations are based, including the differences in ionic strengths, the low concentration of the vanadium used, and the fact that some reduction takes place during the growth experiment to be changing the concentrations somewhat of the VCit and PV complexes. Regardless, our general considerations demonstrate that VCit and PV complexes should form as predicted by the speciation analysis (Pettersson et al., 1983; Ehde et al., 1989; Selling et al., 1994) and reflect the equilibrium mixture observed by NMR analysis.

## Discussion

Vanadium derivatives have been considered as therapeutic agents since 1899 (Lyonnet et al., 1899; Crans et al., 2018). Recent studies demonstrate that vanadium compounds and salts are effective antidiabetic agents (Sakurai, 2002; Thompson and Orvig, 2006; Kiss et al., 2008; Smith et al., 2008; Thompson et al., 2009; Willsky et al., 2011, 2013; Crans, 2015; Trevino et al., 2015; Boulmier et al., 2017; Levina and Lay, 2017b) but their therapeutic properties have been expanded for these materials to be anticancer agents (Lu and Zhu, 2011; Leon et al., 2017; Medina et al., 2017; Crans et al., 2018), immunotherapy agents (Lu and Zhu, 2011; Selman et al., 2018) as well as treatment for other diseases (Zwolak, 2014; Pessoa et al., 2015). Many of the compounds are coordination complexes, which form vanadate during processing after administration (Thompson et al., 2009; Willsky et al., 2011). Considering the ease with which vanadium undergoes redox chemistry (Crans et al., 2010), it is known that some of the vanadium is converted from V(V) to lower oxidation states upon administration (Thompson et al., 2009; Willsky et al., 2011). Furthermore, studies were carried out on the salts prior to investigation of coordination complexes (Sakurai et al., 1990; Goldfine et al., 2000; Willsky et al., 2001, 2013; Smith et al., 2008). The biological activity of homo-oxovanadates have been of considerable interest considering the varied biological effects depending on the specific vanadium species (Sakurai et al., 1990; Willsky et al., 2001; Postal et al., 2016). Information on cellular uptake by vanadium species is of interest, and the studies presented here compare the effect of monomeric vanadate and decavanadate. Such direct comparison is important to understand the interconversion between these species, and despite the many reports in the literature (reviewed in Aureliano and Crans, 2009), no similar study showing data for direct comparison between the two different species have been reported.

Cellular studies have been reported in different types of organisms that consider the uptake of vanadate. Willsky reported studies with yeast (Willsky et al., 1984b, 1985; Crans et al., 2002), and concluded that V_1_ was the species entering the cells; however, upon entry processing took place; forming decavanadate in the lysosomes, and forming V(IV) through redox processes. The treatment of the yeast cells with vanadate solutions containing mainly vanadate monomer and oligomers is evidenced by ^51^V NMR spectroscopy and EPR spectroscopy. These studies were very elegant and truly important, because they documented the formation of decavanadate in cells from monomeric vanadate for the first time (Willsky et al., 1984b). Recently, Zakrzewska and Zivic reported a series of studies in fungi exploring the uptake of both vanadate and V(IV) using ^51^V and EPR spectroscopy, as well as polarographic studies (Zizic et al., 2013, 2016; Hadzibrahimovic et al., 2017). The initial ^51^V NMR studies on *Phycomyce blakesleeanus* mycelium were supplemented with polarography, which allowed for the assignment of the signal at −535 ppm accumulating inside the fungi cells to V_1_ even though the pH of the system was defined by HEPES buffer at 7.2 where V_1_ generally has a peak position at a higher frequency (Zizic et al., 2016). The authors attribute the signal shifting to intracellular complexation of V_1_. Alternatively, these observations are consistent with a hydrophobic environment having been reported in some cases to alter composition and speciation and impact the shifting of observed signals. Combined, these studies showed convincingly that the cell wall is not responsible for reduction of vanadate (Hadzibrahimovic et al., 2017) and these studies also confirm that tetrameric vanadate is not able to enter the cells (Zizic et al., 2016) as reported in yeast as well (Crans et al., 2002) (Willsky et al., 1984a,b.)

Cellular studies in mammalian systems have also been carried out, and many of these studies are linked to the stability and speciation of vanadium compounds in blood (Delgado et al., 2005; Zhang et al., 2006; Li et al., 2009; Jakusch et al., 2011, 2014; Willsky et al., 2013; Sanna et al., 2014, 2017; Jakusch and Kiss, 2017). Although bacterial cells do not contain blood, they do infect mammals and thus the vanadium chemistry in blood is relevant to bacterial infections as well. These studies include detailed investigations into various complexes, and projections based on studies in model systems what would be observed in mammalian systems and *in vivo*. Although such studies remain model studies, they are important because they begin to provide a view of how it will look like in the *in vivo* cells. Studies were also reported by Garner and coworkers using ^1^H NMR spin echo and ^51^V NMR spectroscopy to characterize the uptake of vanadate and subsequent reduction to form V(IV) in erythrocytes (Garner et al., 1997). The reduction of the vanadate was attributed to glutathione as demonstrated in a time-dependent response using both ^51^V NMR and ^1^H spin echo NMR (Garner et al., 1997). The addition of the blocking agent 4,4′-diisothio-cyanatostilbene-2,2b-disulfonic acid (DIDS) specifically blocks the anion transporter preventing the vanadate from entering the erythrocytes. Adding the blocker prevented vanadate entering, and the depletion of glutathione was arrested. Thus, depletion of intracellular gluthathione could be correlated with entering vanadate and the authors proposed that the reduced vanadium [presumably V(IV)] formed complexed with cellular components, because no or little V(IV) formed (Garner et al., 1997).

These studies stand in direct contrast to studies in which decavanadate is associated with proteins and have been characterized by X-ray crystallography (Winkler et al., 2017). However, consideration of both solution and solid state is necessary to characterize both the effects of decavanadate and simple oxovanadates. The studies shown here demonstrate three important findings. First, the fact that decavanadate is a potent inhibitor of growth of two mycobacterial species and more so than monomeric vanadate, which is a known potent phosphatase inhibitor (Crans, 2015; Mclauchlan et al., 2015). Indeed, phosphatase inhibition has recently been reported by V_10_ of the Leishmania acid phosphatase as well as earlier reports (Aureliano and Crans, 2009; Dorsey et al., 2018). Second, the fact that monomeric vanadate is not as potent inhibitor for *M. tb and M. smeg*. Third, these studies also demonstrate that under conditions in which the decavanadate would remain stable, in the presence of *Mycobacterium* spp. the decavanadate undergoes hydrolysis. These combined observations suggest that some type of mechanism that involves the bacterium (or something excreted by the bacterium) interacting with the decavanadate and causes the conversion to oxovanadates. This is particularly interesting because similar response is not observed in the media without bacteria.

## Conclusion

The studies presented demonstrate that decavanadate (V_10_) inhibits the growth of two mycobacterial species, *M. tb* and *M. smeg*, whereas the oxovanadates prepared from NaVO_3_ inhibit growth with 10-100-fold less potency. The inhibition was observed in media containing citrate and phosphate, resulting in the formation of a VCit complex and PV complexes. However, neither of these complexes appeared to interfere with the observed inhibition for V_10_ and the inhibition by V_1_ was still 10-100-fold less. The greater inhibitory selectivity of V_10_ (or a component of V_10_) result is important because it demonstrates that simple vanadium salts can have different effects on cells, and that a vanadium compound other than the potent phosphatase inhibitor, monomeric vanadate (V_1_), can inhibit growth. Importantly, these studies suggest that other oxometalates may have the ability to inhibit growth of mycobacteria and other pathogens.

In addition, these studies demonstrated that mycobacteria or some component excreted by the mycobacteria which catalyzes the hydrolysis of V_10_ and the same effect was not observed in media in the absence of growing cells. This process is particularly interesting because it implies that the V_10_ interacts with the some cell component, possibly through some mechanism involving V_10_ delivering a V-atom to such a cell component. This type of mechanism is novel and further investigation into such process will be of general interest and important for the mode of action of POMs in biological systems.

## Author contributions

NS carried out the ^51^V NMR studies, prepared the figures and wrote part of the manuscript. ZA carried out the growth studies with vanadate and decavanadate and recorded some of the NMR spectra. ZA also collected the experiments with the colors of the various media. HM did the speciation calculations. SK and DCri advised and oversaw the biological studies. DCra combined the chemical and biological components of the work, advised and oversaw the NMR studies and wrote the manuscript with the assistance of NS. All authors contributed to the editing of the manuscript.

### Conflict of interest statement

The authors declare that the research was conducted in the absence of any commercial or financial relationships that could be construed as a potential conflict of interest.

## Supplementary material

More figures are included in the supplementary materials showing additional spectra supporting the results presented in the manuscript and include NMR experiments, growth experiments and additional information on the speciation calculations.
