# Supplementary material for: Decavanadate Inhibits Mycobacterial Growth More Potently Than Other Oxovanadates
Source: Front Chem. 2018 Nov 20;6:519. doi: 10.3389/fchem.2018.00519 (PMC6255961; doi:10.3389/fchem.2018.00519)
Supplement: Supplementary file 1 [file Data_Sheet_1.pdf]

# Decavanadate inhibits mycobacterial growth more potently than other oxovanadates

***Nuttaporn Samart[1,2], Zeyad Arhouma[1,3], Santosh Kumar[4], Heide A. Murakami[1], Dean C. Crick[2,4] and Debbie C. Crans,[1,2]****

[1]Department of Chemistry Colorado State University, Fort Collins, Colorado 80523, USA
[2]Department of Chemistry, Rajabhat Rajanagarindra University, Chachoengsao 24000, Thailand
[3]Cell & Molecular Biology Program, Colorado State University, Fort Collins, Colorado 80523, USA
[4]Department of Microbiology, Immunology and Pathology, Colorado State University, Fort Collins, Colorado 80523, USA

Table of Content

**Figure S1.** The 100 mM sodium orthovanadate ($Na_3VO_4$) p 2

**Figure S2.** The 10 mM sodium metavanadate ($NaVO_3$) at varying pH values p 3

**Figure S3.** Spectra of $V_1$ and $V_{10}$ in the absence and presence of media p 4

**Figure S4.** Spectra of $V_{10}$ at various conditions (pH, media and reference) p 5

**Figure S5.** Spectra of $V_1$ at various conditions (pH, media and reference) p 6

**Figure S6.** The growth curve of *M. tb* by decavanadate p 7

**Figure S7.** The growth curve of *M. tb* by metavanadate p 8

**Figure S8.** The growth curve of *M. smeg* by decavanadate p 9

**Figure S9.** The growth curve of *M. smeg* by metavanadate p 10

**Table 1. Experimental data monitoring color and pH valus of growth studies p. 11**

**Table 2. Data in all the studies done testing the changesin pH values of cultures p.14**

**Detailed experimental data from heating (or not) supernatant of media in which mycobacteria were grown added $V_{10}$ or $V_1$** p. 15

**Figure S10.** The evaluation of the speciation at different $V_{10}$ p 26

**Figure S11.** The evaluation of the speciation at $V_{10}$ only species present p 27

Tables 3-5 Details in Speciation Calculations p 28

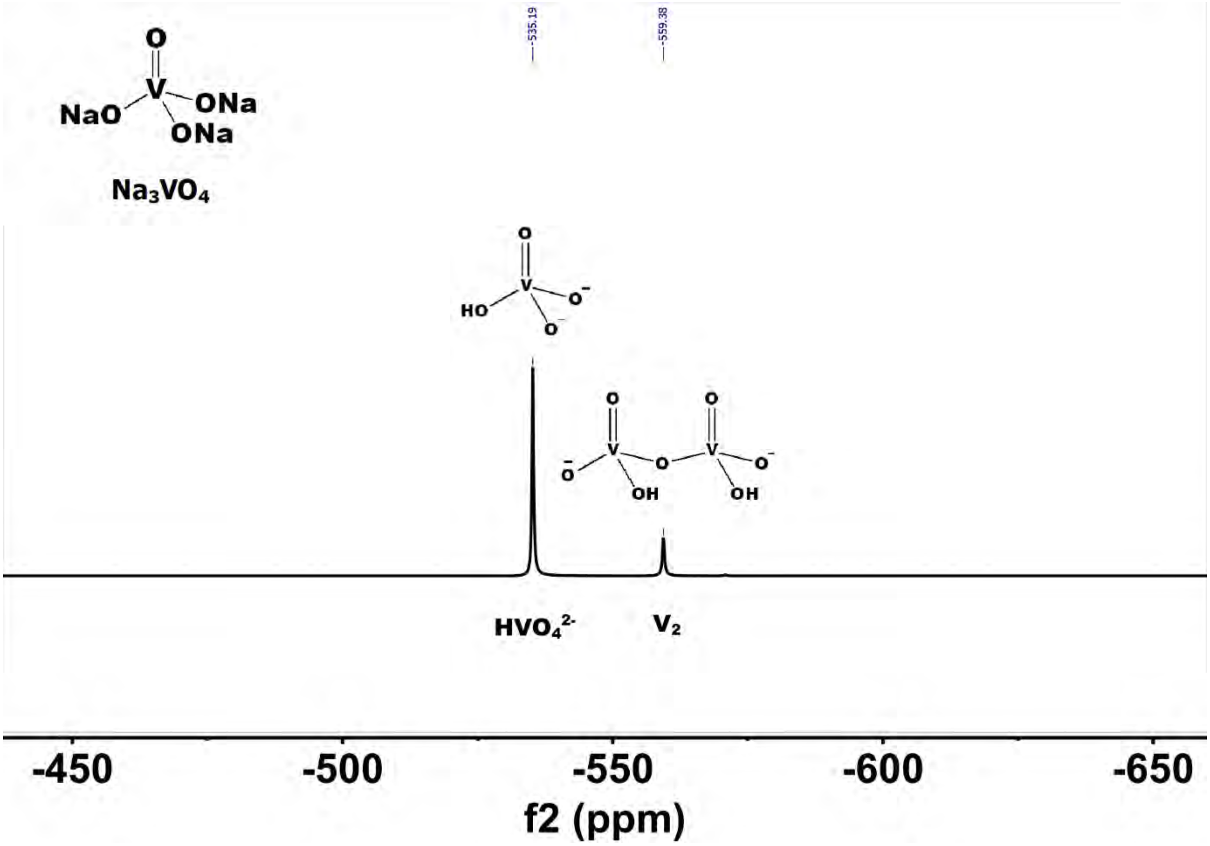


**Figure S1.** The 100 mM sodium orthovanadate ($Na_3VO_4$) at pH 12.96 original solution showing two signals monomeric vanadate ($HVO_4^{2-}$ abbreviated $V_1$) strong peak and dimeric vanadate (abbreviated $V_2$), at -535 ppm and -559 ppm, respectively. In this work use $Na_3VO_4$ for external reference (Crans, 1994).

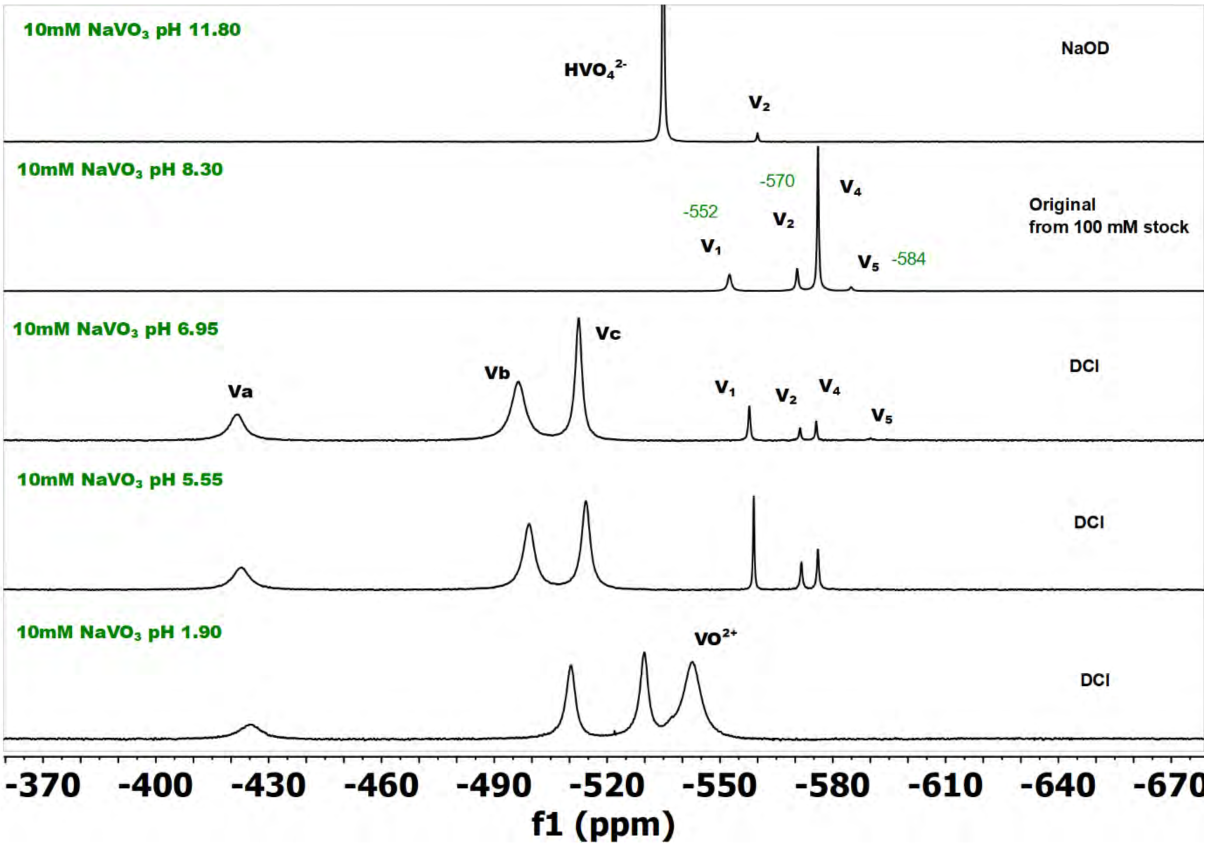


**Figure S2.** The 10 mM sodium metavanadate ($NaVO_3$) at varying pH values adjust by using 6 M DCl and 6 M NaOD solution. The original solution of $NaVO_3$ colorless at pH 8.30 spectra show signal monomer ($V_1$), dimer ($V_2$), tetramer ($V_4$) and pentamer ($V_5$). In acid pH 5.55-6.95 get decavanadate form ($V_{10}$) (The decavanadate have 3 difference V atom as Va, Vb and Vc signal show -420 to -530 ppm) and at low pH $NaVO_3$ change charge lose proton transfer. $NaVO_3$ in basic pH 11.80 adjust by NaOD show signal 2 peaks assignment monomer vanadate form $HVO_4^{2-}$ at -535 ppm and dimer form at -559 ppm

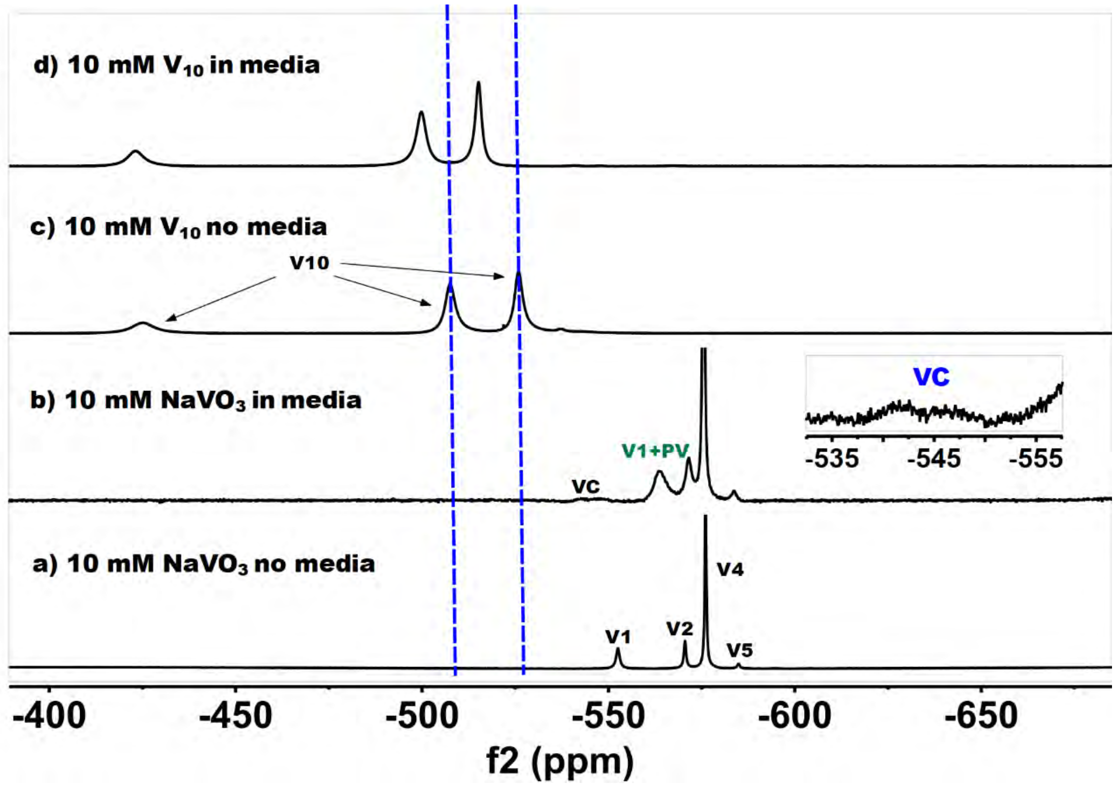


**Figure S3.** Spectra of a) $NaVO_3$ in aqueous solution (no media), b) $NaVO_3$ in media and c) $V_{10}$ in aqueous solution (no media) and d) $V_{10}$ in media. Key for signals in the case of the VCit complex two different broad peaks at -537 and -550 ppm are shown. In the case of PV the rapid exchange between $V_1$ and PV lead to only one signal at -559 ppm including both these compounds.

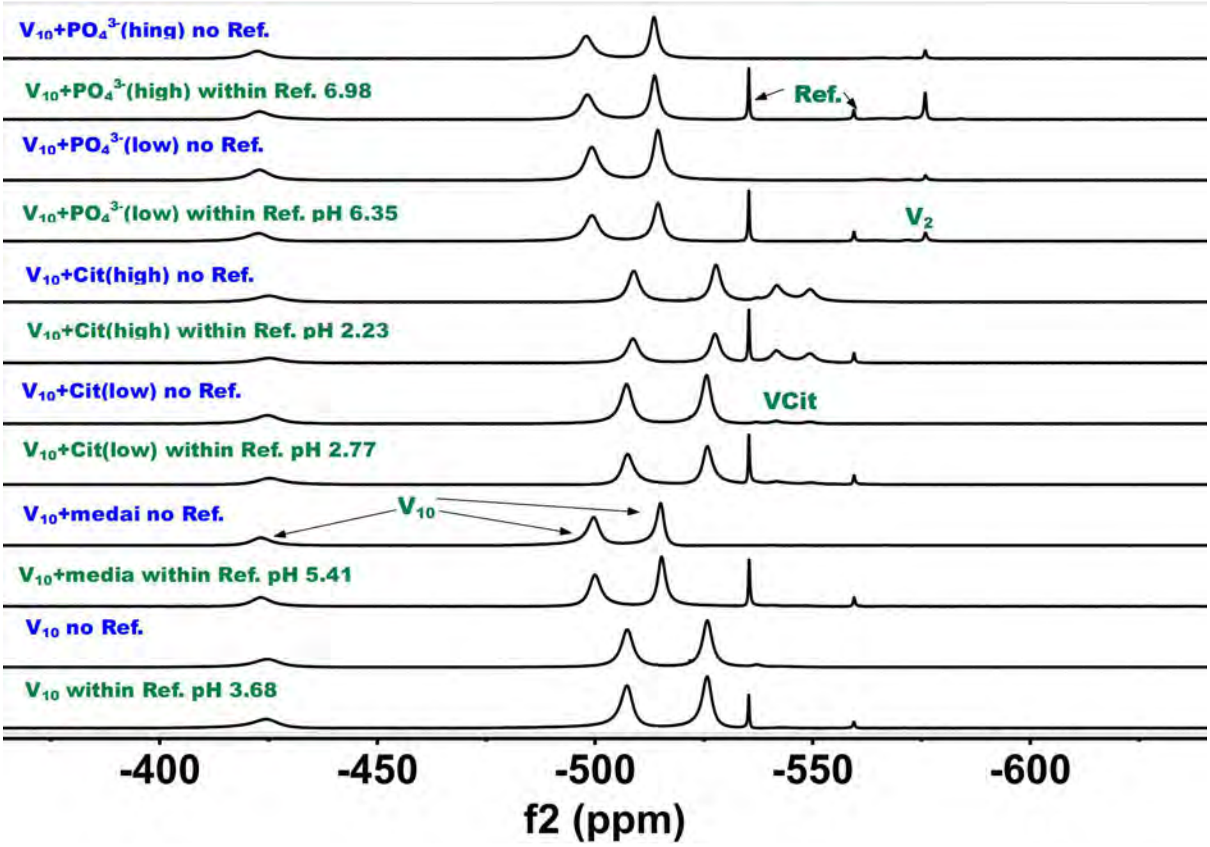


**Figure S4.** Spectra of $V_{10}$ at various conditions including different pH values and in the presence and absence of media and reference. Signals are labeled; selected spectra from this figure was used to make Fig. 2 in manuscript.

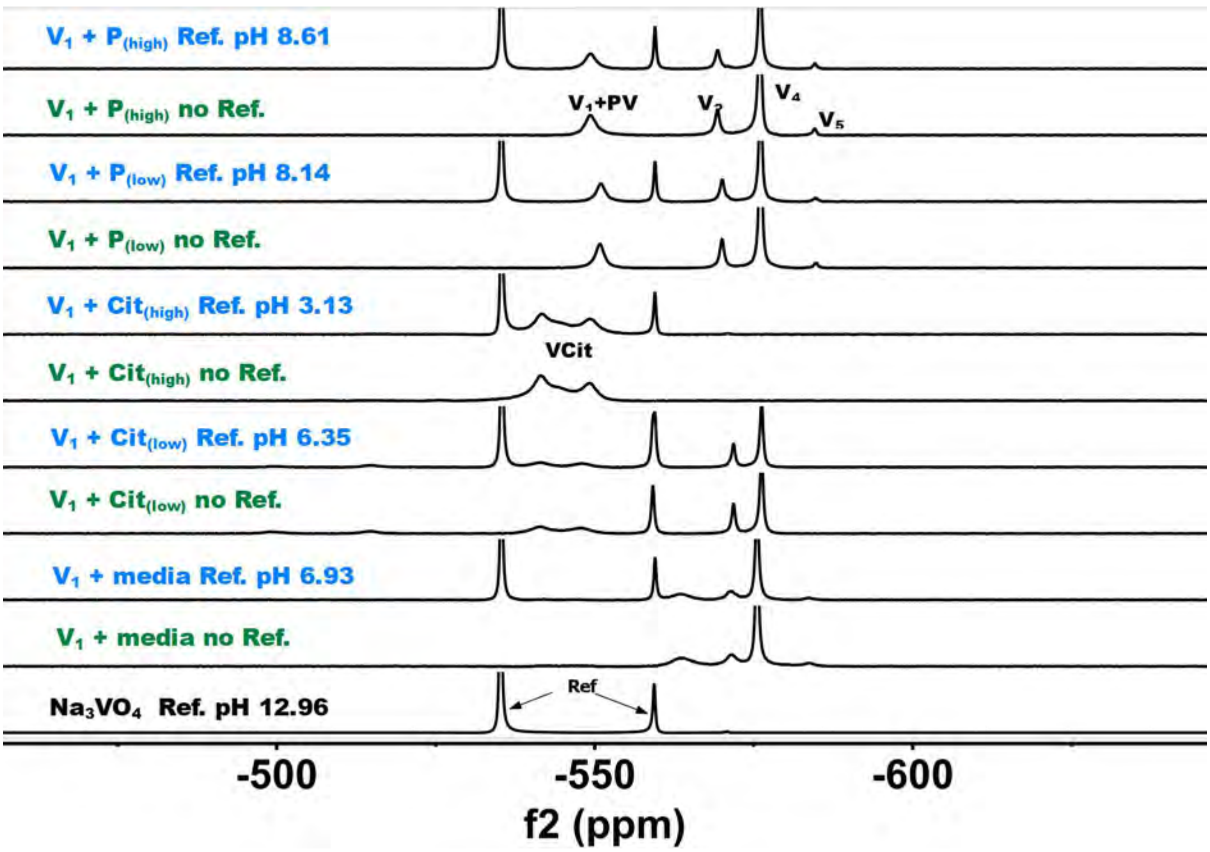


**Figure S5.** Spectra of $NaVO_3$ in the form of $V_1$ at neutral and basic pH values in the presence and absence of media. Oxovanadate signals are labeled as well as VCit and PV. Selected spectra from this figure was used to prepare Fig. 3 in manuscript.

**Figure S6.** The growth curve is shown for treatment with decavanadate (prepared from a 100 mM orange decavanadate solution, 1.0 M V-atoms) on *M.tb.*

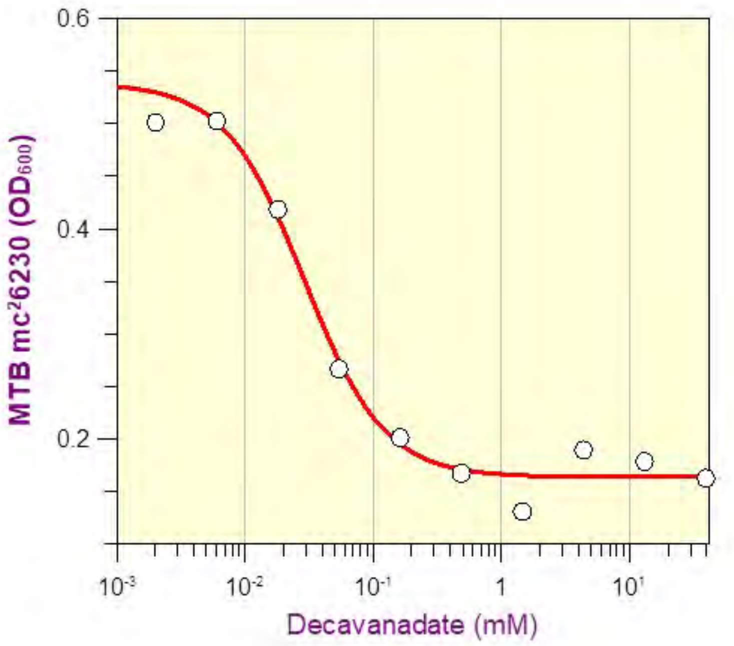


| Parameter | Value | Std. Error |
|---|---|---|
| Y Range | 0.3751 | 0.0216 |
| $IC_{50}$ | 0.0290 | 0.0050 |
| Slope factor | 1.3939 | 0.2755 |
| Background | 0.1636 | 0.0106 |

| Decavanadate ($V_{10}$) (mM) | $Mc^2$ 6320 (OD600) |
|---|---|
| 40.0000 | 0.1617 |
| 13.3333 | 0.1777 |
| 4.4444 | 0.1887 |
| 1.4815 | 0.1300 |
| 0.4938 | 0.1663 |
| 0.1646 | 0.2003 |
| 0.0549 | 0.2657 |
| 0.0183 | 0.4177 |
| 0.0061 | 0.5020 |
| 0.0020 | 0.5007 |
| 0.0000 | 0.5623 |

**Figure S7.** The growth curve is shown for treatment with metavanadate (prepared from a 40 mM colorless metavanadate solution, 0.40 M V-atoms) on *M.tb.*

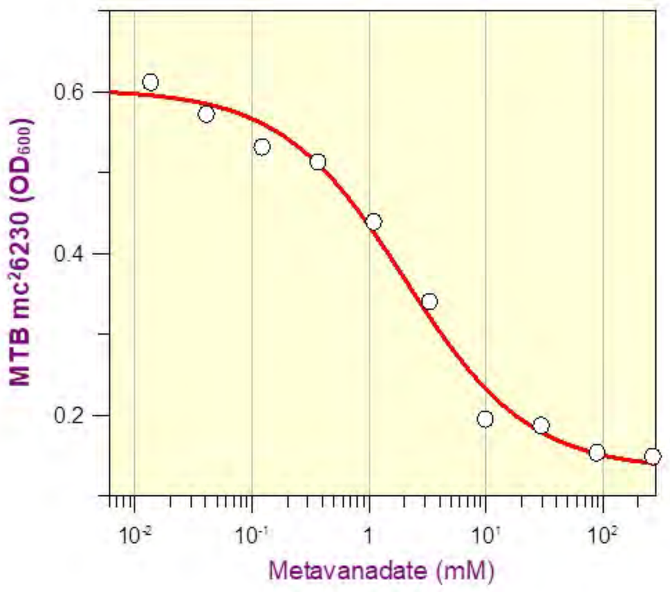


| Parameter | Value | Std. Error |
|---|---|---|
| Y Range | 0.4715 | 0.0299 |
| $IC_{50}$ | 2.0319 | 0.4385 |
| Slope factor | 0.8209 | 0.1320 |
| Background | 0.1318 | 0.0212 |

| Metavanadate ($V_1$) (mM) | $Mc^2$ 6320 (OD600) |
|---|---|
| 270.0000 | 0.1480 |
| 90.0000 | 0.1527 |
| 30.0000 | 0.1860 |
| 10.0000 | 0.1943 |
| 3.3333 | 0.3397 |
| 1.1111 | 0.4383 |
| 0.3704 | 0.5123 |
| 0.1235 | 0.5310 |
| 0.0412 | 0.5717 |
| 0.0137 | 0.6110 |
| 0.0000 | 0.6163 |

**Figure S8.** The growth curve is shown for treatment with decavanadate (prepared from a 100 mM orange decavanadate solution (1.0 M V-atoms) on *M.smeg*.

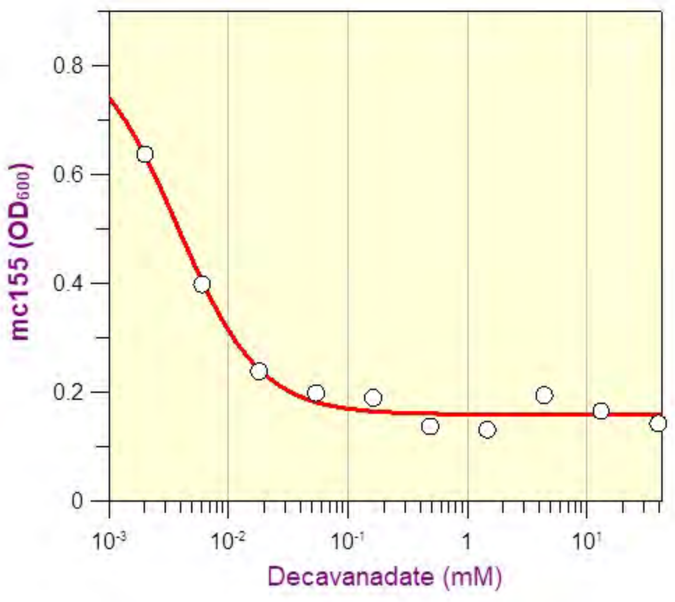


| Parameter | Value | Std. Error |
|---|---|---|
| Y Range | 0.6922 | 0.0255 |
| $IC_{50}$ | 0.0037 | 0.0004 |
| Slope factor | 1.2641 | 0.1501 |
| Background | 0.1591 | 0.0095 |

| **Decavanadate ($V_{10}$) (mM)** | **$Mc^2$ 155 (OD600)** |
|---|---|
| 40.0000 | 0.1413 |
| 13.3333 | 0.1640 |
| 4.4444 | 0.1933 |
| 1.4815 | 0.1297 |
| 0.4938 | 0.1360 |
| 0.1646 | 0.1887 |
| 0.0549 | 0.1970 |
| 0.0183 | 0.2370 |
| 0.0061 | 0.3973 |
| 0.0020 | 0.6367 |
| 0.0000 | 0.8500 |

**Figure S9.** The growth curve is shown for treatment with metavanadate (prepared from a 40 mM colorless metavanadate solution, 0.40 M V-atoms) on *M. smeg.*

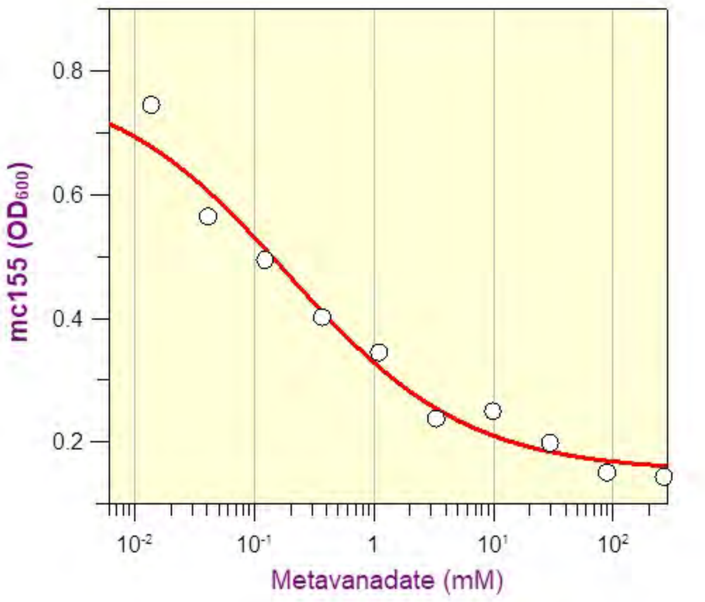


| Parameter | Value | Std. Error |
|---|---|---|
| Y Range | 0.6399 | 0.0514 |
| $IC_{50}$ | 0.1888 | 0.0706 |
| Slope factor | 0.5801 | 0.1103 |
| Background | 0.1518 | 0.0297 |

| Metavanadate ($V_1$) (mM) | $Mc^2$ 155 (OD600) |
|---|---|
| 270.0000 | 0.1427 |
| 90.0000 | 0.1497 |
| 30.0000 | 0.1970 |
| 10.0000 | 0.2483 |
| 3.3333 | 0.2367 |
| 1.1111 | 0.3440 |
| 0.3704 | 0.4007 |
| 0.1235 | 0.4933 |
| 0.0412 | 0.5637 |
| 0.0137 | 0.7443 |
| 0.0000 | 0.7723 |

**Table 1. $V_1$ & $V_{10}$ added to 7H9 Media (in the absence of bacteria) at different time points (both 3.3 and 10 mM $V_1$ (colorless right) and $V_{10}$ (yellow/oranges); pH are measured and shown in table. No green color is developing in either samples – indicating no redox in samples.**

## 1) 0 hr

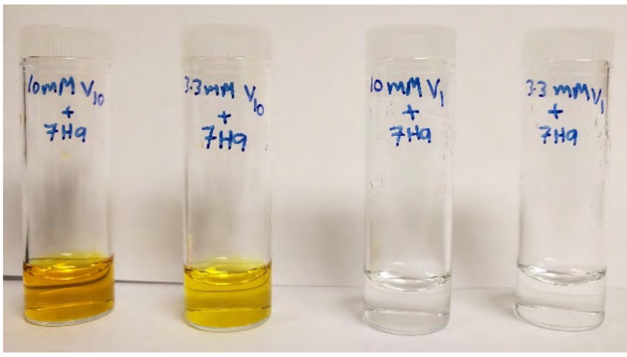


| | pH | |
|---|---|---|
| | 10mM | 3.3mM |
| $V_1$ | 7.42 | 7.39 |
| $V_{10}$ | 7.30 | 7.33 |

## 2) 1 hr

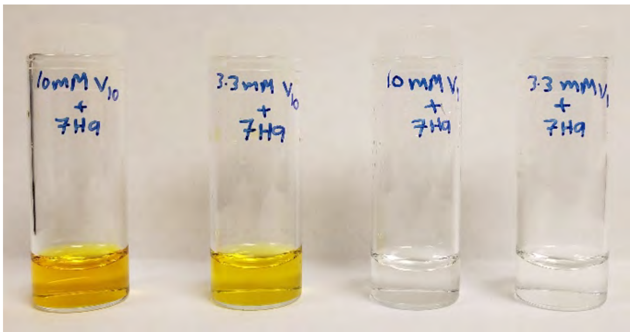


| | pH | |
|---|---|---|
| | 10mM | 3.3mM |
| $V_1$ | 7.04 | 7.01 |
| $V_{10}$ | 6.78 | 6.88 |

## 3) 5 hrs

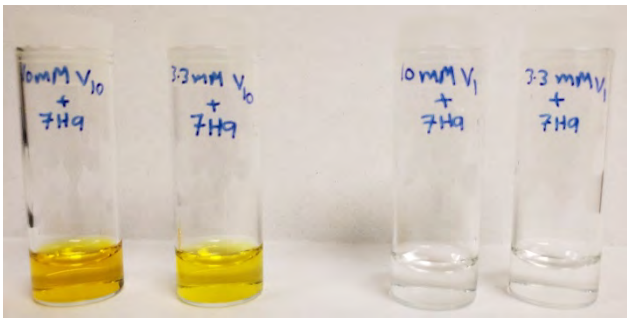


| | pH | |
|---|---|---|
| | 10mM | 3.3mM |
| $V_1$ | 7.10 | 7.05 |
| $V_{10}$ | 6.63 | 6.82 |

## 4) 24 hrs

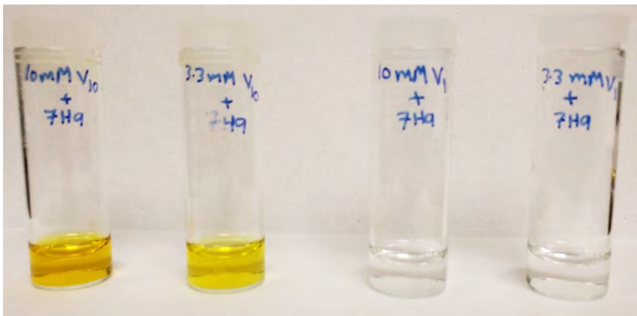


| | pH | |
|---|---|---|
| | 10mM | 3.3mM |
| $V_1$ | 7.02 | 6.97 |
| $V_{10}$ | 6.47 | 6.50 |

**Table 1 (continued). $V_1$ (3.3 and 10 mM, colorless right) & $V_{10}$ (3.3 and 10 mM yellow-organic-green) in 7H9 media with *M.smegmatis*. The pH values are measured at different time points and shown in a table. Green color is developing after 5 hours of incubations in the samples containing $V_{10}$ consistent with redox.**

## 1) 0 hr

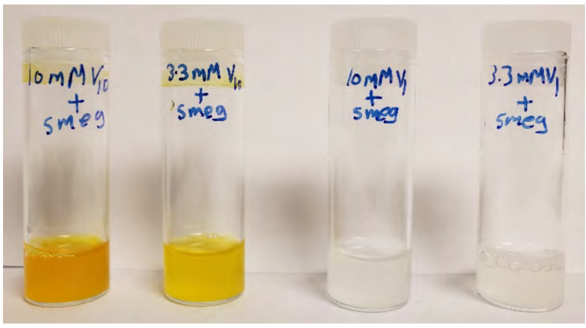


| pH | | |
|---|---|---|
| | 10mM | 3.3mM |
| $V_1$ | 7.32 | 7.30 |
| $V_{10}$ | 7.24 | 7.27 |

## 2) 1 hr

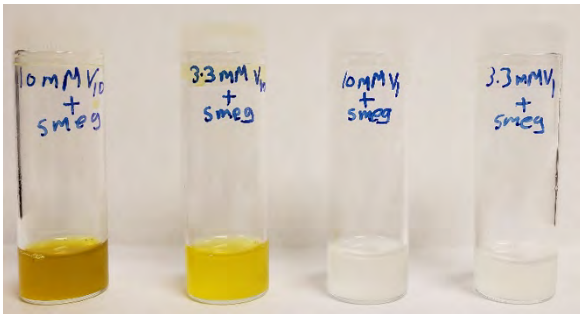


| pH | | |
|---|---|---|
| | 10mM | 3.3mM |
| $V_1$ | 6.89 | 6.84 |
| $V_{10}$ | 6.40 | 6.66 |

## 3) 5 hrs

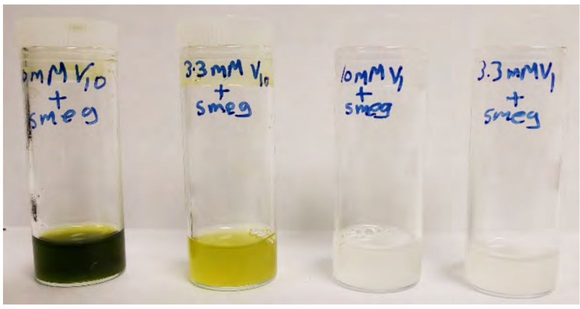


| pH | | |
|---|---|---|
| | 10mM | 3.3mM |
| $V_1$ | 6.83 | 6.78 |
| $V_{10}$ | 6.43 | 6.41 |

## 4) 24 hrs

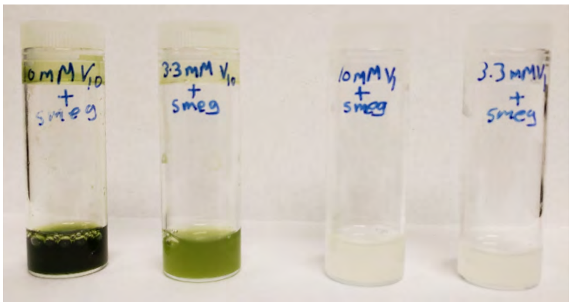


| pH | | |
|---|---|---|
| | 10mM | 3.3mM |
| $V_1$ | 6.54 | 6.58 |
| $V_{10}$ | 6.22 | 6.20 |

Tabel 1 continued. $V_1$ (colorless, right) & $V_{10}$ (yellow, orange, green) (3.3 and 10 mM) added to supernatant in which *M.smeg* had been grown. The solution color are shown after treatment with $V_{10}$ and $V_1$ solutions at different time points of V incubation (with supernatant no cells) and the pH is shown in the table.

1) 0 hr

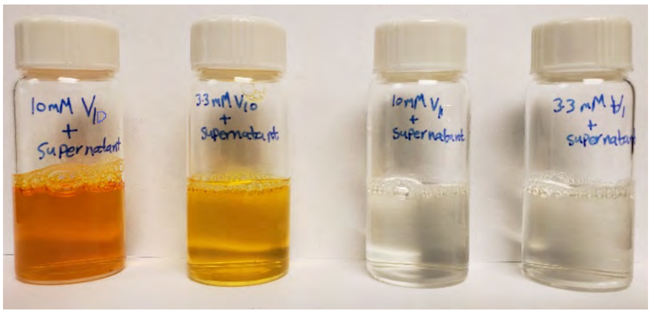


| pH | | |
|---|---|---|
| | 10mM | 3.3mM |
| $V_1$ | 6.86 | 6.82 |
| $V_{10}$ | 6.75 | 6.79 |

2) 1 hr

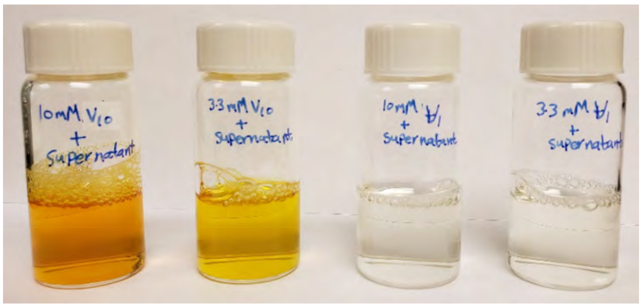


| pH | | |
|---|---|---|
| | 10mM | 3.3mM |
| $V_1$ | 6.85 | 6.80 |
| $V_{10}$ | 6.37 | 6.63 |

3) 5 hrs

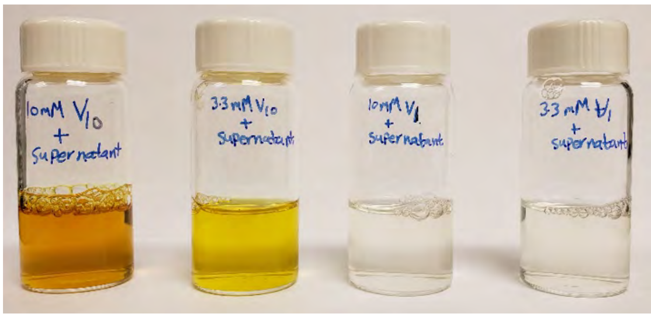


| pH | | |
|---|---|---|
| | 10mM | 3.3mM |
| $V_1$ | 6.88 | 6.83 |
| $V_{10}$ | 6.17 | 6.26 |

4) 24 hrs

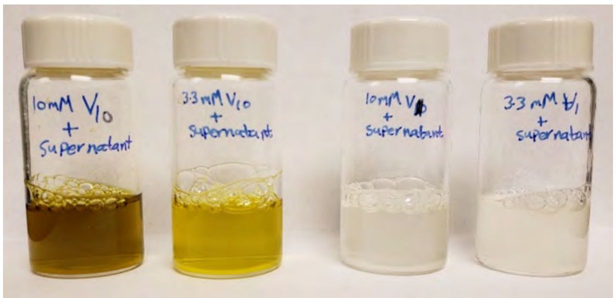


| pH | | |
|---|---|---|
| | 10mM | 3.3mM |
| $V_1$ | 6.67 | 6.57 |

## Table 2. The pH values measured in 7H9 media, *M. Smeg* cultures and supernatant after M. smeg. growi after incubation with V-samples

| Time incubated | $V_{10}$ or $V_1$ - 7H9 media | | | | $V_{10}$ or $V_1$ – *M. smeg* in 7H9 media | | | | $V_{10}$ or $V_1$ – supernatant after *M. smeg* growth (cells removed) | | | |
|---|---|---|---|---|---|---|---|---|---|---|---|---|
| **V-treatment** | **$V_{10}$ (mM)** | | **$V_1$ (mM)** | | **$V_{10}$ (mM)** | | **$V_1$ (mM)** | | **$V_{10}$ (mM)** | | **$V_1$ (mM)** | |
| **V-species concentration** | **10.0 mM** | **3.3 mM** | **10.0 mM** | **3.3 mM** | **10.0 mM** | **3.3 mM** | **10.0 mM** | **3.3 mM** | **10.0 mM** | **3.3 mM** | **10.0 mM** | **3.3 mM** |
| **0** | **7.30** | **7.33** | **7.42** | **7.39** | **7.24** | **7.27** | **7.32** | **7.30** | **6.75** | **6.79** | **6.86** | **6.82** |
| **1** | **6.78** | **6.88** | **7.04** | **7.01** | **6.40** | **6.66** | **6.89** | **6.84** | **6.37** | **6.63** | **6.85** | **6.80** |
| **5** | **6.63** | **6.82** | **7.10** | **7.05** | **6.43** | **6.41** | **6.83** | **6.78** | **6.17** | **6.26** | **6.88** | **6.83** |
| **24** | **6.47** | **6.50** | **7.02** | **6.97** | **6.22** | **6.20** | **6.54** | **6.58** | **6.07** | **6.12** | **6.67** | **6.57** |

**In the following session we investigated the nature of the material that is excreted by the bacterium and causing the reduction.**

**That is we wondered if the material acted differently if heated. The idea here is that if the material was a protein such as the tyrosine phosphatases heating the supernantant solution would change the effects of the protein on the $V_{10}$. Since this was not observed (see data below) it is more likely that the material created by the *M. smeg.* is a siderophore.**

# $V_1$ & $V_{10}$ + Heated Supernatant at different time points

## 5) 0 hr

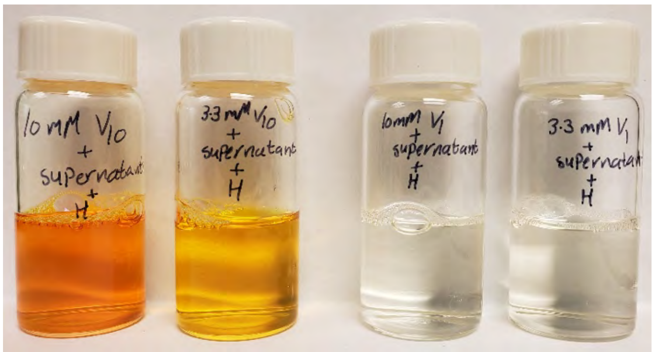


| pH | | |
|---|---|---|
| | 10mM | 3.3mM |
| $V_1$ | 6.82 | 6.75 |
| $V_{10}$ | 6.67 | 6.70 |

## 6) 1 hr

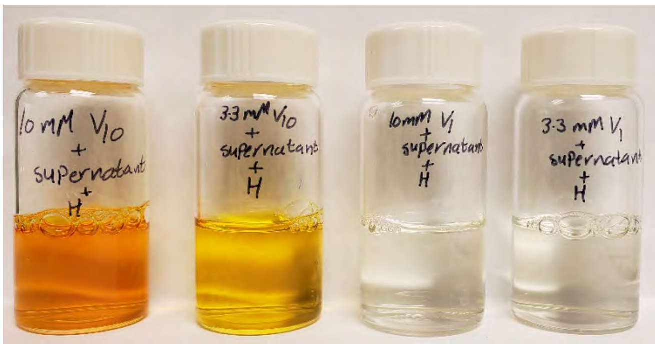


| pH | | |
|---|---|---|
| | 10mM | 3.3mM |
| $V_1$ | 6.86 | 6.82 |
| $V_{10}$ | 6.47 | 6.65 |

## 7) 5 hrs

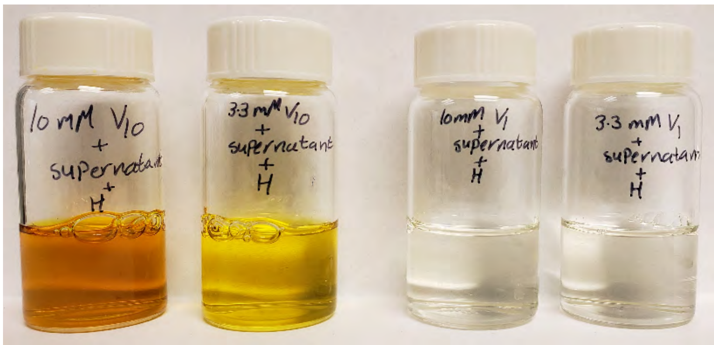


| pH | | |
|---|---|---|
| | 10mM | 3.3mM |
| $V_1$ | 6.82 | 6.77 |
| $V_{10}$ | 6.02 | 6.14 |

## 8) 24 hrs

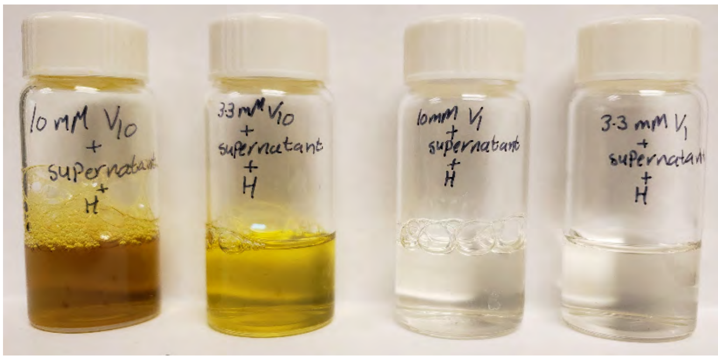


| pH | | |
|---|---|---|
| | 10mM | 3.3mM |
| $V_1$ | 6.81 | 6.76 |
| $V_{10}$ | 6.04 | 6.09 |

## $V_1$ & $V_{10}$ + UnHeated Supernatant at different time points

### 5) 0 hr

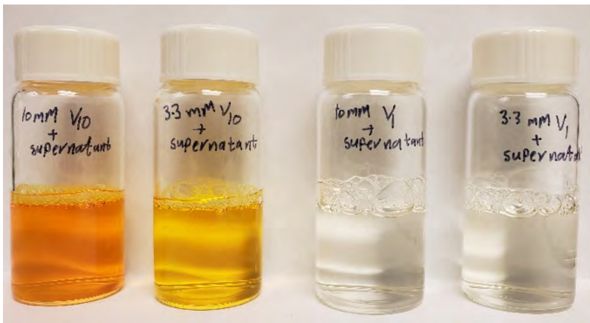


| pH | | |
|---|---|---|
| | 10mM | 3.3mM |
| $V_1$ | 6.88 | 6.84 |
| $V_{10}$ | 6.83 | 6.82 |

### 6) 1 hr

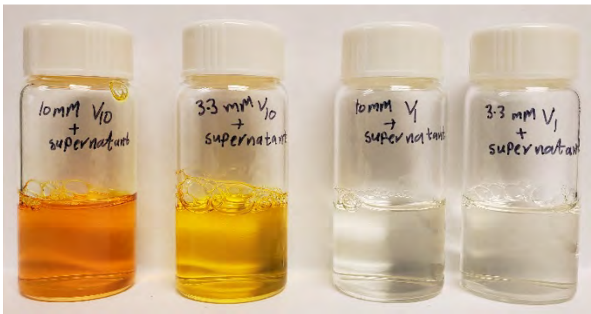


| pH | | |
|---|---|---|
| | 10mM | 3.3mM |
| $V_1$ | 6.86 | 6.81 |
| $V_{10}$ | 6.58 | 6.71 |

### 7) 5 hrs

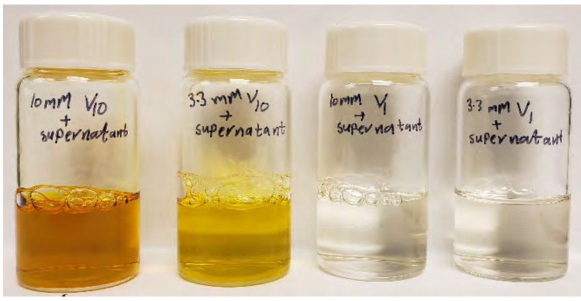


| pH | | |
|---|---|---|
| | 10mM | 3.3mM |
| $V_1$ | 6.83 | 6.77 |
| $V_{10}$ | 5.99 | 6.13 |

### 1) 24 hrs

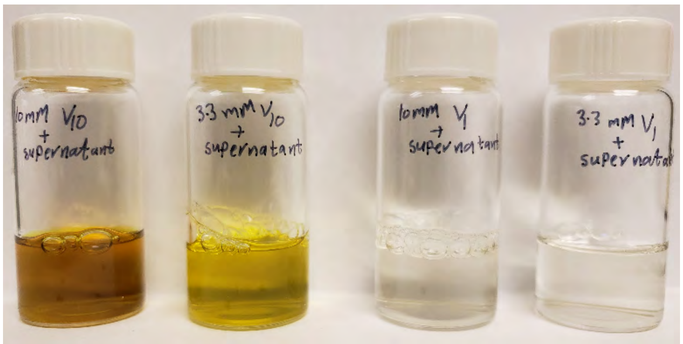


| pH | | |
|---|---|---|
| | 10mM | 3.3mM |
| $V_1$ | 6.82 | 6.75 |
| $V_{10}$ | 6.00 | 6.08 |

**48 hrs**

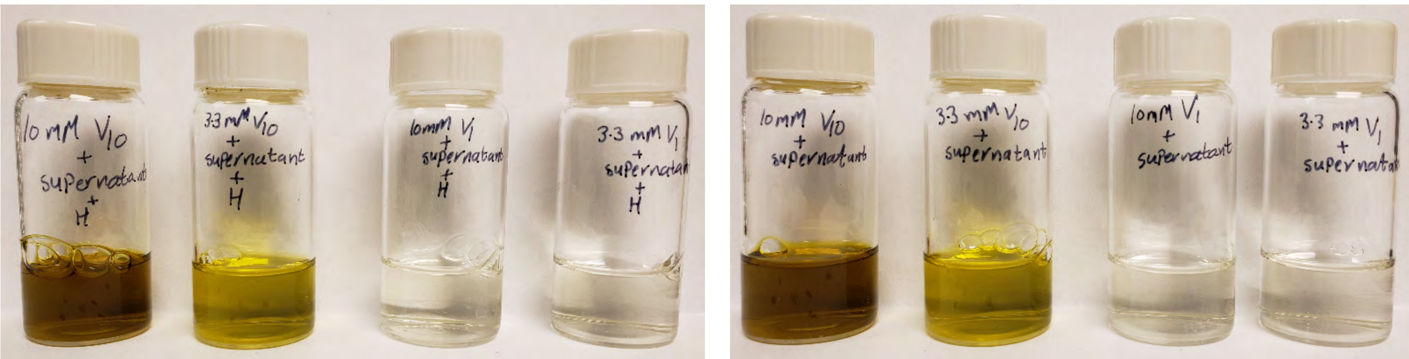


# Resazurin Assay

**Test done to make sure their is no bacteria in the supernatant**

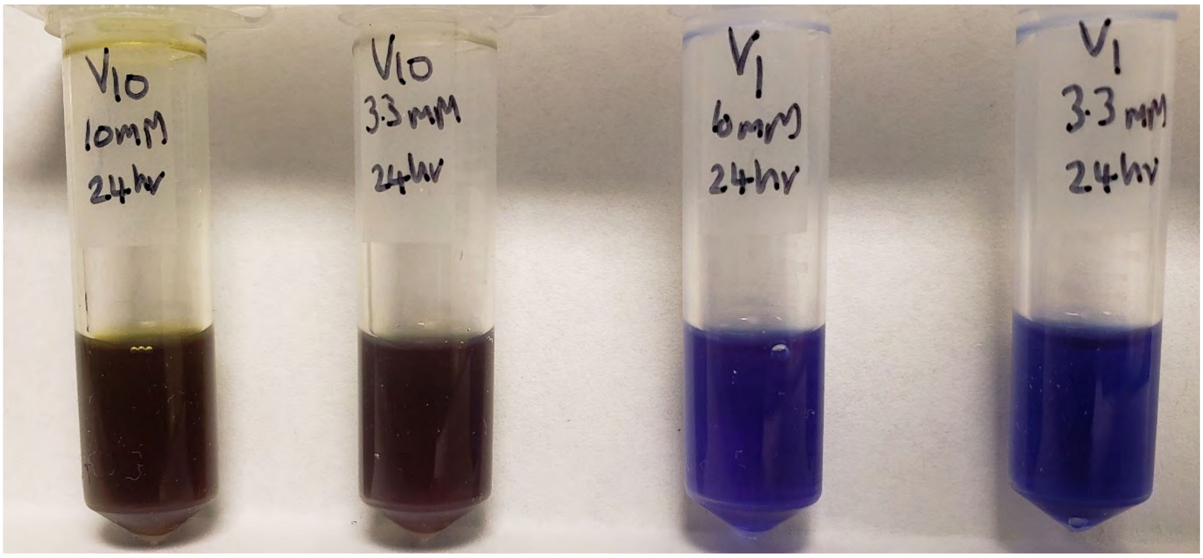

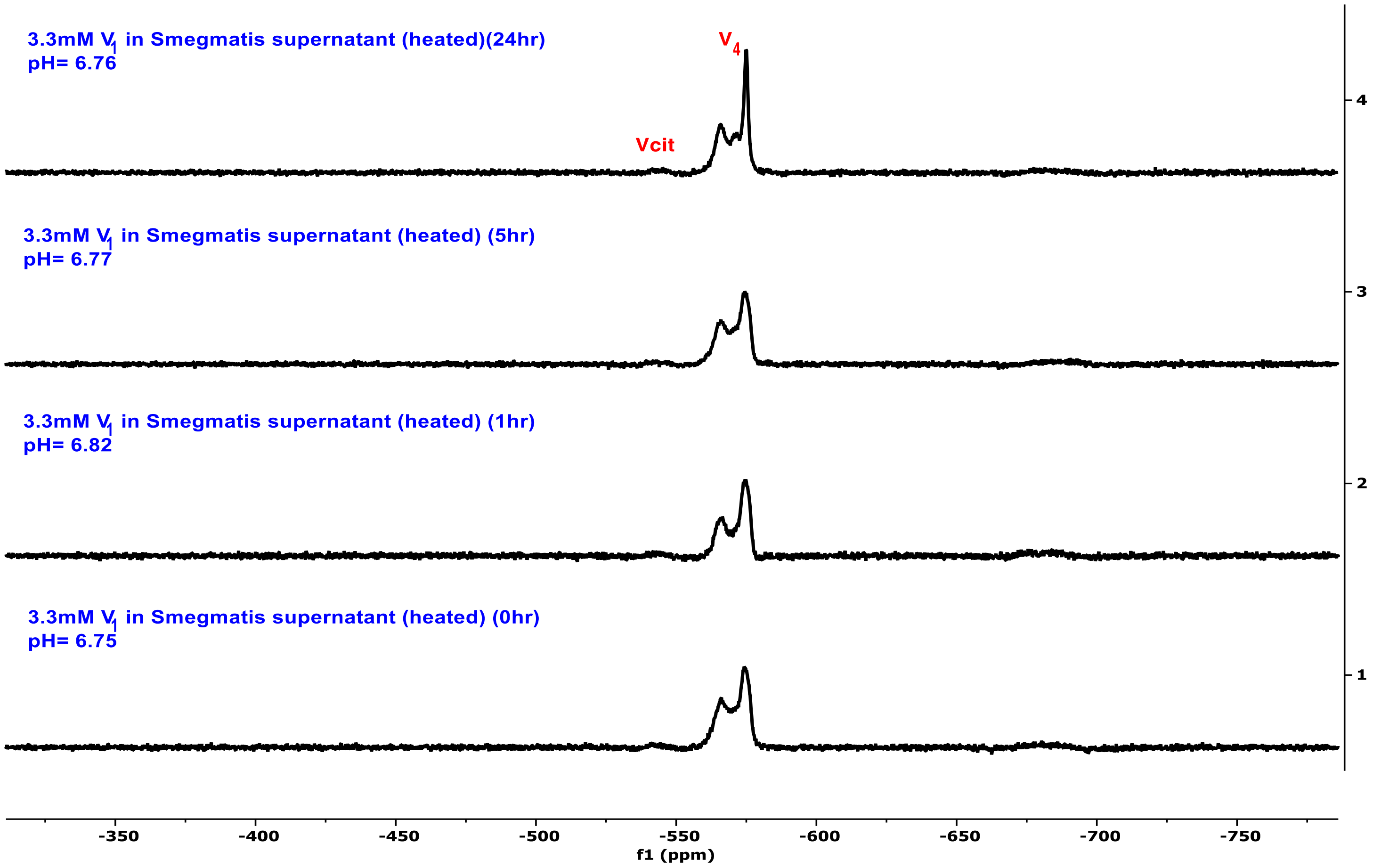
3.3mM V1 in Smegmatis supernatant (heated)(24hr)
pH= 6.76
V4
Vcit
3.3mM V1 in Smegmatis supernatant (heated) (5hr)
pH= 6.77
3.3mM V1 in Smegmatis supernatant (heated) (1hr)
pH= 6.82
3.3mM V1 in Smegmatis supernatant (heated) (0hr)
pH= 6.75
-350 -400 -450 -500 -550 -600 -650 -700 -750
f1 (ppm)

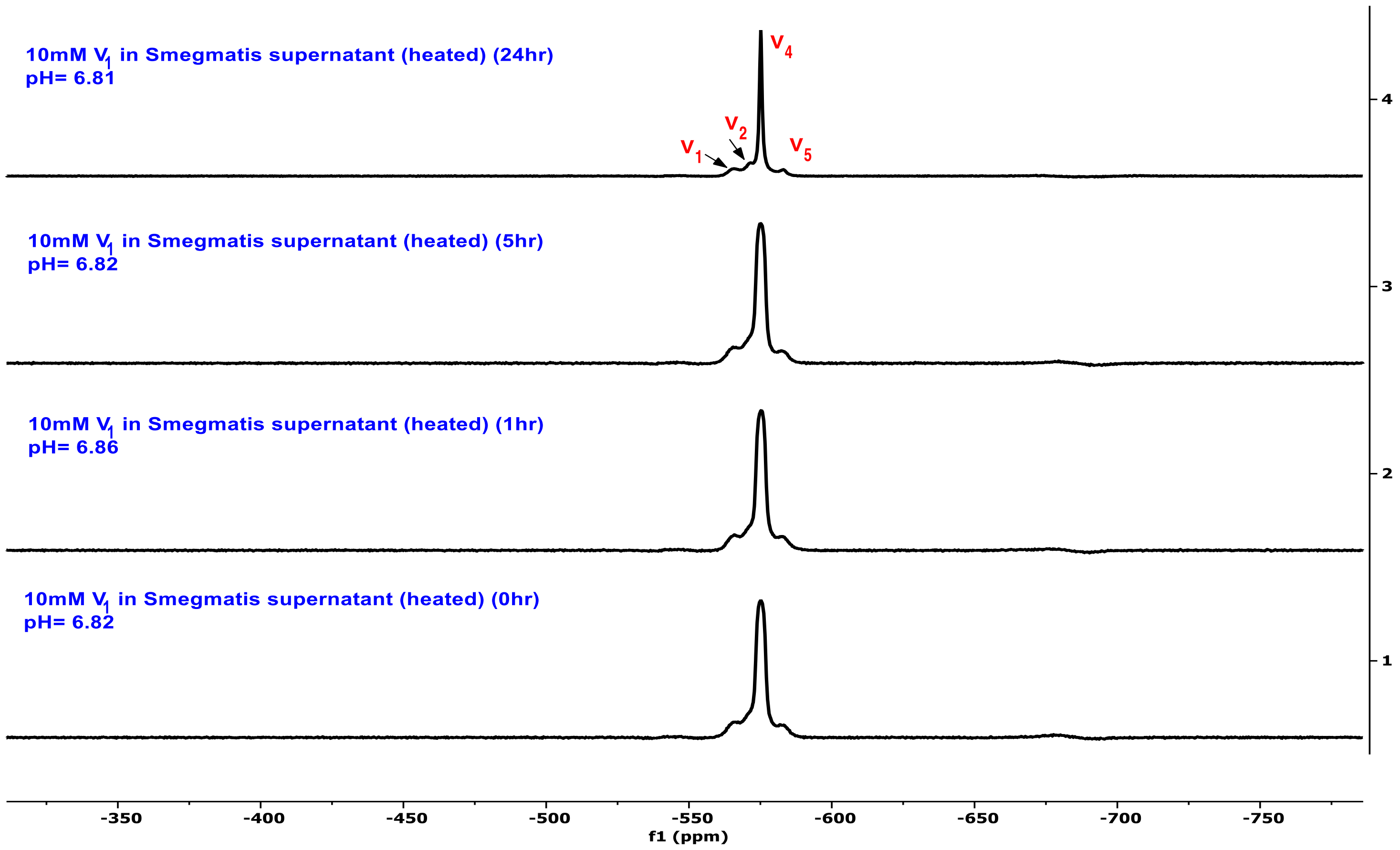
10mM V1 in Smegmatis supernatant (heated) (24hr)
pH= 6.81
V4
V2
V1
V5
10mM V1 in Smegmatis supernatant (heated) (5hr)
pH= 6.82
10mM V1 in Smegmatis supernatant (heated) (1hr)
pH= 6.86
10mM V1 in Smegmatis supernatant (heated) (0hr)
pH= 6.82
4
3
2
1
-350
-400
-450
-500
-550
-600
-650
-700
-750
f1 (ppm)

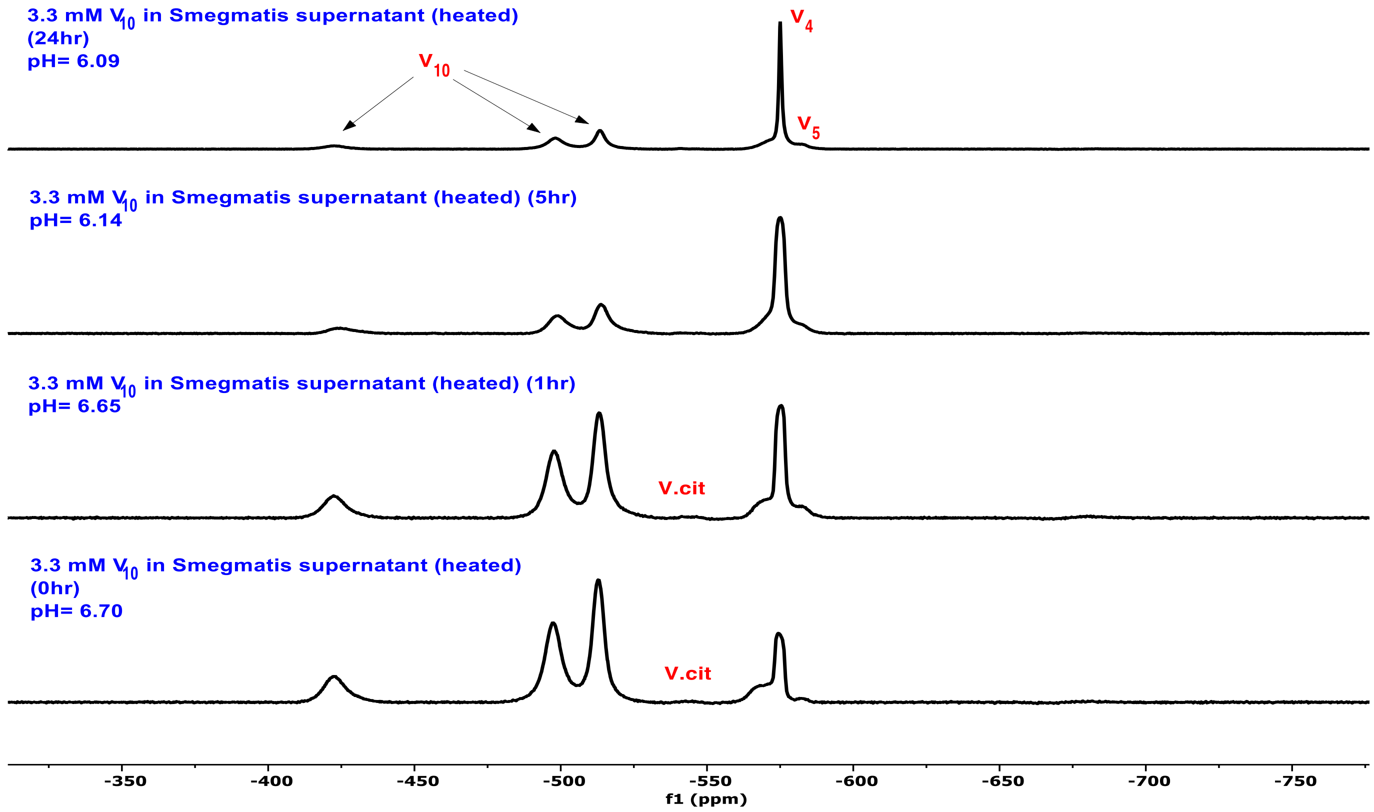
3.3 mM V10 in Smegmatis supernatant (heated)
(24hr)
pH= 6.09
V10
V4
V5
3.3 mM V10 in Smegmatis supernatant (heated) (5hr)
pH= 6.14
3.3 mM V10 in Smegmatis supernatant (heated) (1hr)
pH= 6.65
V.cit
3.3 mM V10 in Smegmatis supernatant (heated)
(0hr)
pH= 6.70
V.cit
-350
-400
-450
-500
-550
-600
-650
-700
-750
f1 (ppm)

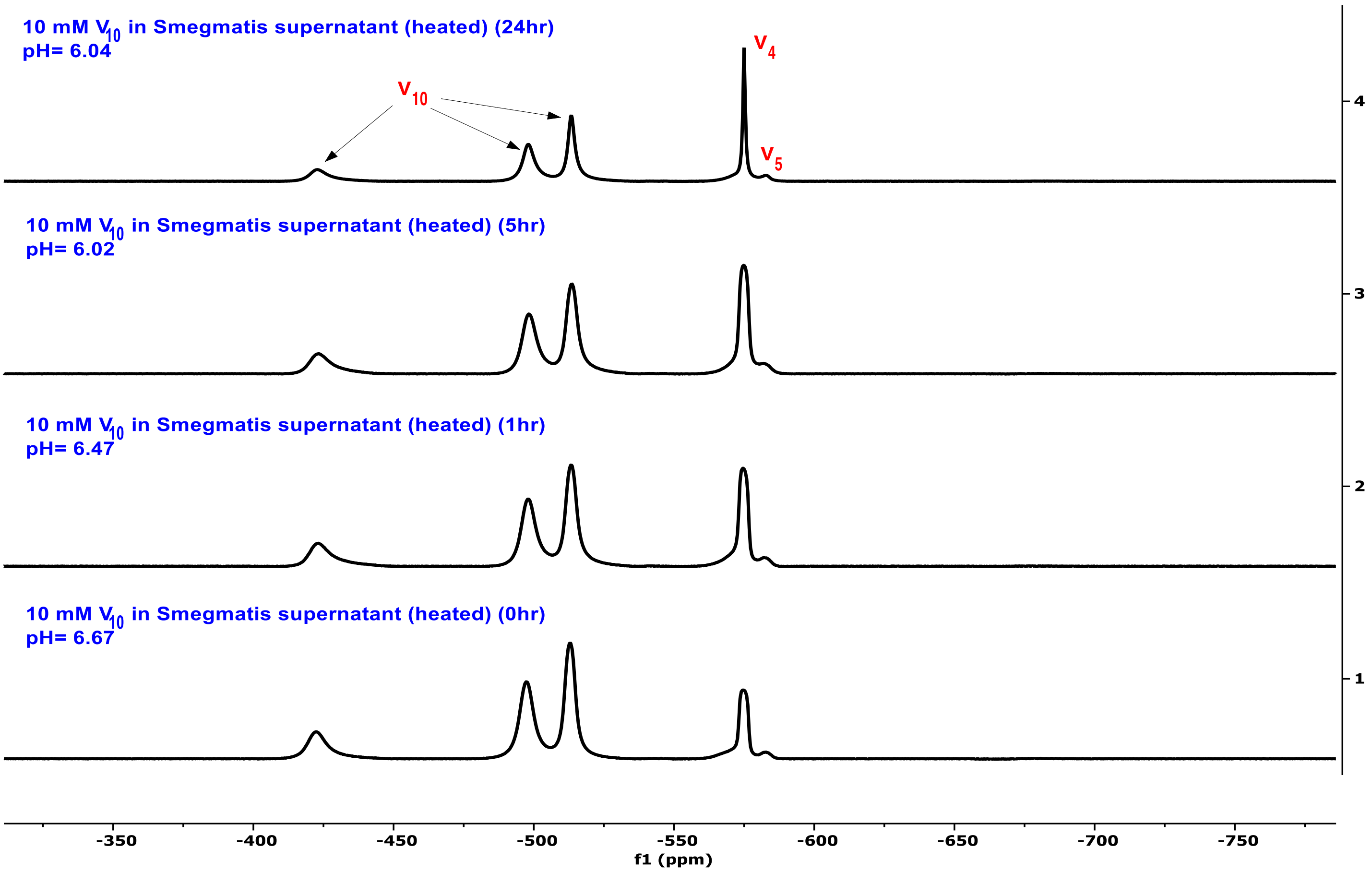
10 mM V10 in Smegmatis supernatant (heated) (24hr)
pH= 6.04
V10
V4
V5
10 mM V10 in Smegmatis supernatant (heated) (5hr)
pH= 6.02
10 mM V10 in Smegmatis supernatant (heated) (1hr)
pH= 6.47
10 mM V10 in Smegmatis supernatant (heated) (0hr)
pH= 6.67
4
3
2
1
-350
-400
-450
-500
-550
-600
-650
-700
-750
f1 (ppm)

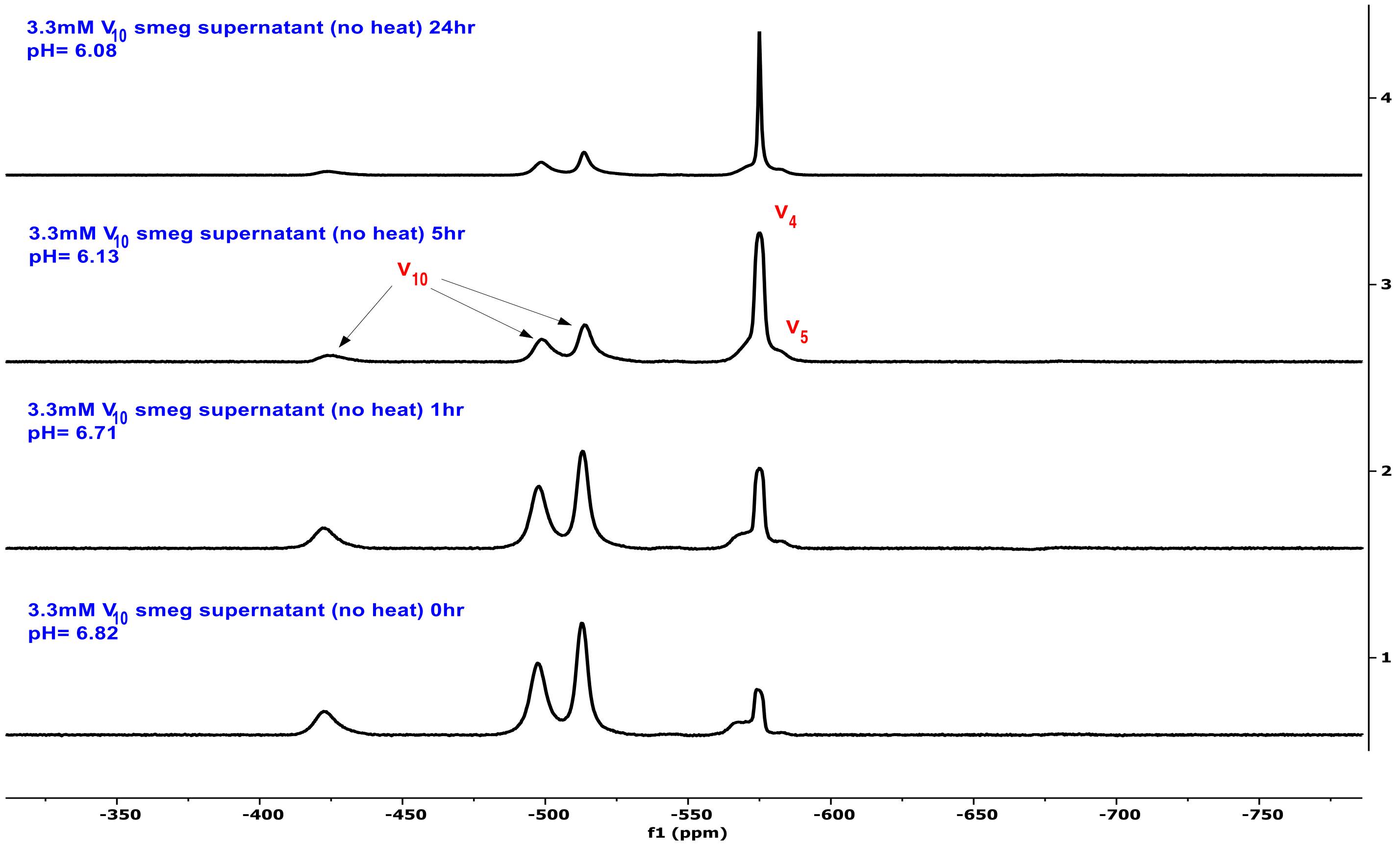

3.3mM V10 smeg supernatant (no heat) 24hr
pH= 6.08
3.3mM V10 smeg supernatant (no heat) 5hr
pH= 6.13
3.3mM V10 smeg supernatant (no heat) 1hr
pH= 6.71
3.3mM V10 smeg supernatant (no heat) 0hr
pH= 6.82
V4
V10
V5
4
3
2
1
-350
-400
-450
-500
-550
-600
-650
-700
-750
f1 (ppm)

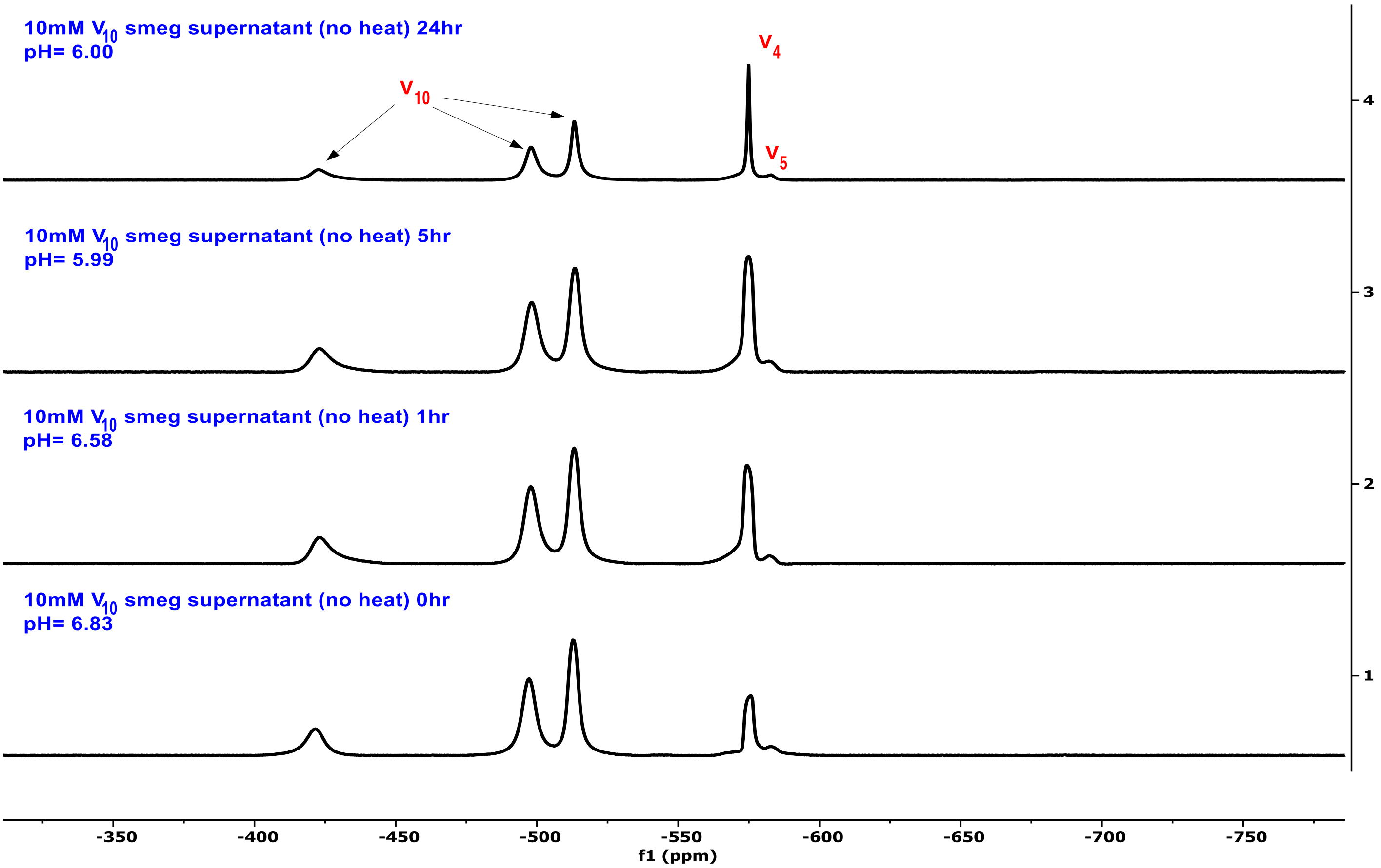
10mM $V_{10}$ smeg supernatant (no heat) 24hr
pH= 6.00
$V_{10}$
$V_4$
$V_5$
10mM $V_{10}$ smeg supernatant (no heat) 5hr
pH= 5.99
10mM $V_{10}$ smeg supernatant (no heat) 1hr
pH= 6.58
10mM $V_{10}$ smeg supernatant (no heat) 0hr
pH= 6.83
4
3
2
1
-350
-400
-450
-500
-550
-600
-650
-700
-750
f1 (ppm)

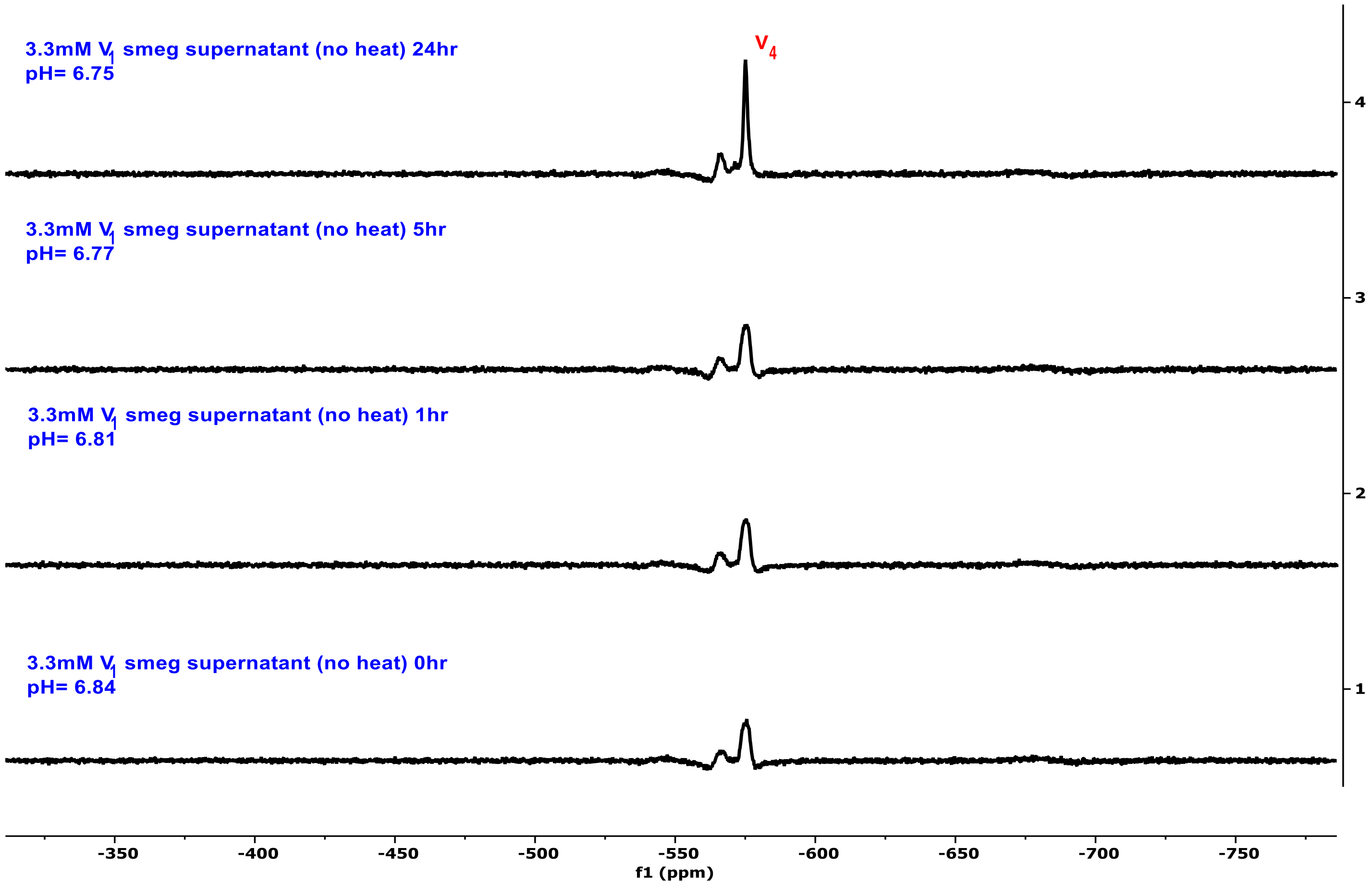
3.3mM V1 smeg supernatant (no heat) 24hr
pH= 6.75
V4
3.3mM V1 smeg supernatant (no heat) 5hr
pH= 6.77
3.3mM V1 smeg supernatant (no heat) 1hr
pH= 6.81
3.3mM V1 smeg supernatant (no heat) 0hr
pH= 6.84
4
3
2
1
-350
-400
-450
-500
-550
-600
-650
-700
-750
f1 (ppm)

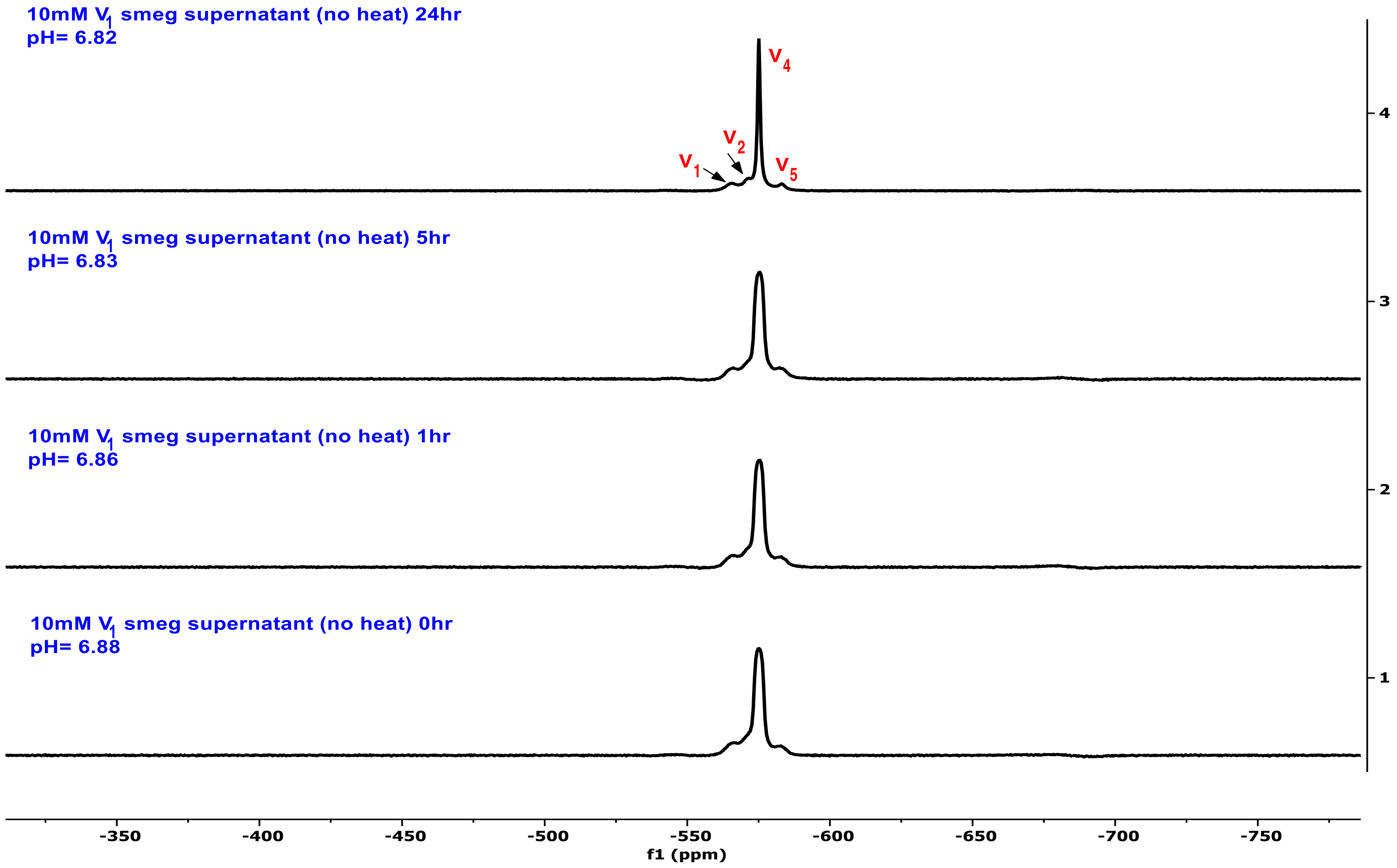
10mM V1 smeg supernatant (no heat) 24hr
pH= 6.82
V4
V2
V1
V5
10mM V1 smeg supernatant (no heat) 5hr
pH= 6.83
10mM V1 smeg supernatant (no heat) 1hr
pH= 6.86
10mM V1 smeg supernatant (no heat) 0hr
pH= 6.88
4
3
2
1
-350
-400
-450
-500
-550
-600
-650
-700
-750
f1 (ppm)

**Figure S10** The evaluation of the speciation at different vanadium concentration using HySS Program (vs 2009) (Alderighi et al., 1999). The speciation diagram shown was calculated at several vanadium (V-atom) concentrations in the presence of 0.48 mM citrate and 24 mM phosphate found in the growth media and a) 5.0 μM vanadate, b) 1.0 mM vanadate c) 3.3 mM vanadate and c) 10 mM vanadate. Note, the concentrations of different species regardless of nuclearity are here shown in terms of V-atoms. These distribution diagrams were used to generate Fig. 7 in the manuscript.

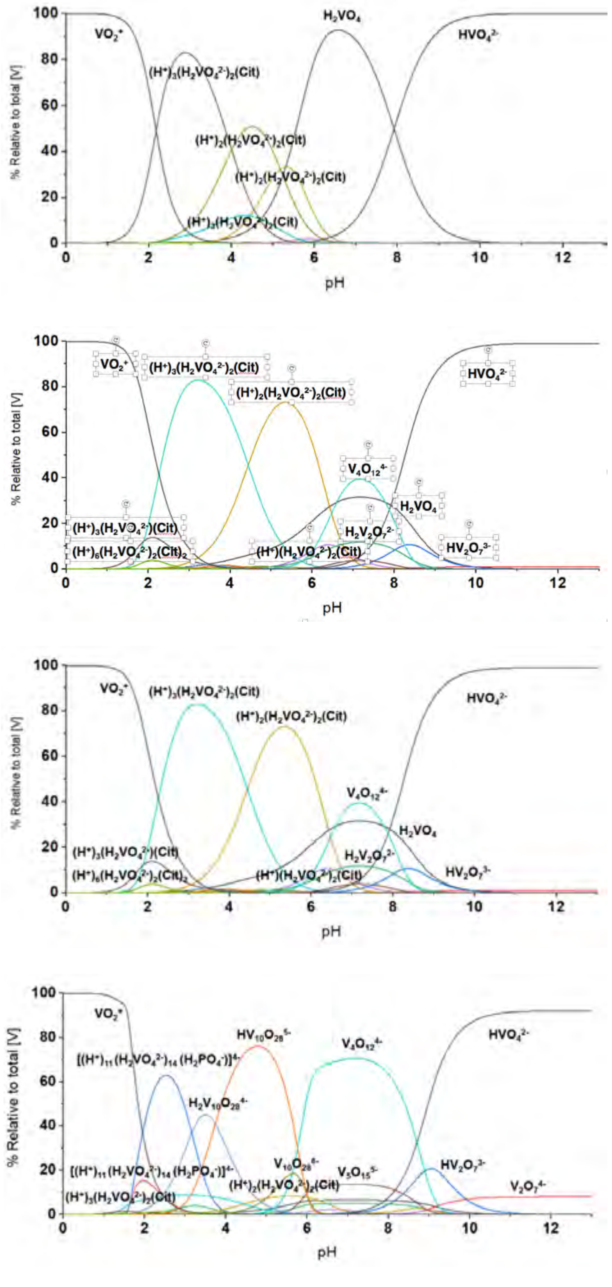

**Figure S11.** The evaluation of the speciation at different $V_{10}$ concentration assuming $V_{10}$ is only species present using HySS Program (vs 2009) (Alderighi et al., 1999). The speciation diagram shown was calculated at several decavanadate concentrations a) 5.0 µM vanadate, b) 3.3 mM vanadate and c) 10 mM vanadate. Note, this speciation assumes decavanadate as the only species in solution to depict speciation at neutral pH where $V_{10}$ only slowly hydrolyze to form other oxometalates.

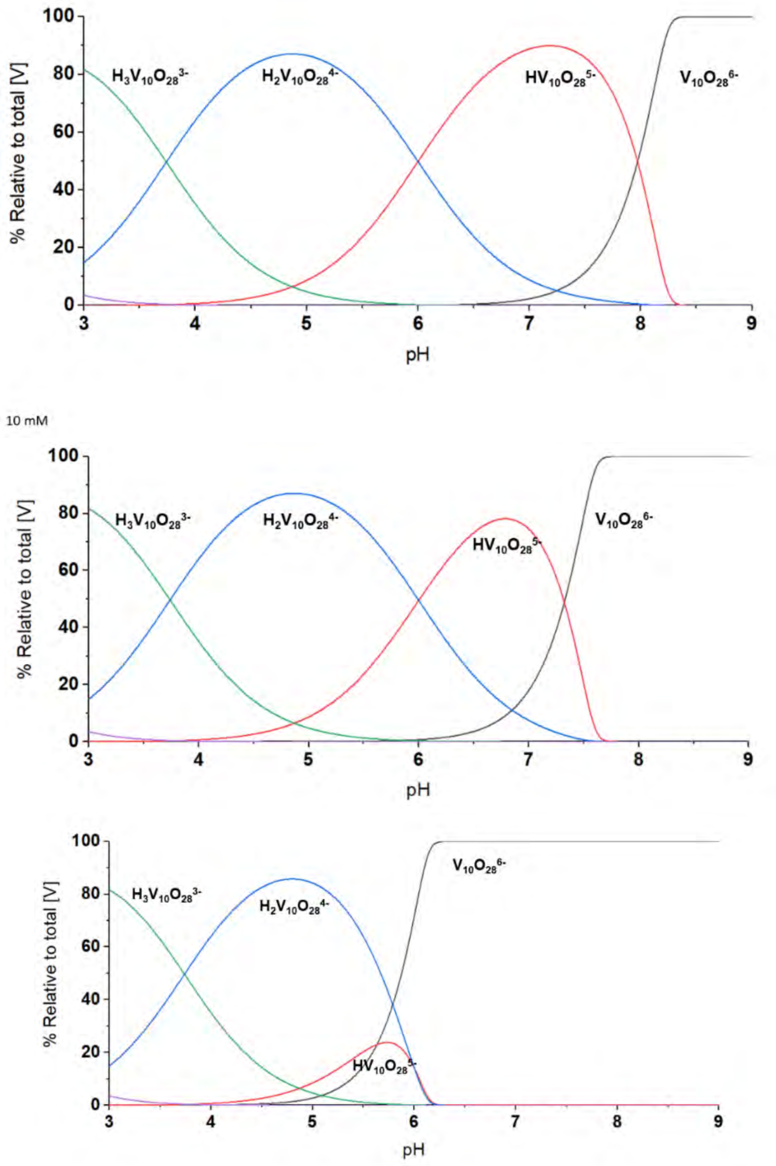

# Speciation Calculations

Additional information is provided regarding the assumptions made for speciation calculations. Species distribution diagrams were calculated by using HySS 2009 software [1]. The citrate and the phosphate concentrations were set as constants to 0.48 mM and 24 mM respectively. The vanadium concentrations that were investigated were concentrations of 5 uM, 1 mM, and 10 mM. The speciation diagrams were constructed using the following equilibrium reactions.

Table 5

| Chemical Reactions | Ref |
|---|---|
| $pH^+ + qH_2VO_4^{2-} \leftrightarrows (H^+)_p\,(H_2VO_4^{2-})_q$ | [2] |
| $pH^+ + qH_2VO_4^{2-} + rC_6H_5O_7^{3-} \leftrightarrows (H^+)_p\,(H_2VO_4^{2-})_q\,(C_6H_5O_7^{3-})_r$ | [3] |
| $pH^+ + qH_2VO_4^{2-} + rH_2PO_4^- \leftrightarrows [(H^+)_p\,(H_2VO_4^{2-})_q\,(H_2PO_4^-)_r]^{p-q-r}$ | [4] |

Table 6. Formation Constants of Vanadate Species in 0.6 NaCl system

| (p, q) | log β | Formula | Extended formula |
|---|---|---|---|
| -1, 1 | -7.92 | $HVO_4^{2-}$ | $(H^+)_{-1}(H_2VO_4^{2-})$ |
| -2, 2 | -15.17 | $V_2O_7^{4-}$ | $(H^+)_{-2}(H_2VO_4^{2-})_2$ |
| -1, 2 | -5.25 | $HV_2O_7^{3-}$ | $(H^+)_{-1}(H_2VO_4^{2-})_2$ |
| 0, 2 | 2.77 | $H_2V_2O_7^{2-}$ | $(H_2VO_4^{2-})_2$ |
| -2, 4 | -8.88 | $V_4O_{13}^{6-}$ | $(H^+)_{-2}(H_2VO_4^{2-})_4$ |
| -1, 4 | 0.22 | $HV_4O_{13}^{5-}$ | $(H^+)_{-1}(H_2VO_4^{2-})_4$ |
| 0, 4 | 10.0 | $V_4O_{12}^{4-}$ | $(H_2VO_4^{2-})_4$ |
| 0, 5 | 12.4 | $V_5O_{15}^{5-}$ | $(H_2VO_4^{2-})_5$ |
| 4, 10 | 52.1 | $V_{10}O_{28}^{6-}$ | $(H^+)_4(H_2VO_4^{2-})_{10}$ |
| 5, 10 | 58.1 | $HV_{10}O_{28}^{5-}$ | $(H^+)_5(H_2VO_4^{2-})_{10}$ |
| 6, 10 | 61.9 | $H_2V_{10}O_{28}^{4-}$ | $(H^+)_6(H_2VO_4^{2-})_{10}$ |
| 7, 10 | 63.5 | $H_3V_{10}O_{28}^{3-}$ | $(H^+)_7(H_2VO_4^{2-})_{10}$ |
| 2, 1 | 6.96 | $VO_2^+$ | $(H^+)_2(H_2VO_4^{2-})_{10}$ |

Data is taken from Pettersson et al. and the formation constants are recalculated for $H_2VO_4^-$ as the vanadium component. [2]

Table 7

| (p, q, r) | log β | Formula | Extended formula |
|---|---|---|---|
| 1, 0, 1 | 5.217 | $Cit^{2-}$ | $(H^+)(Cit^{3-})$ |
| 2, 0, 1 | 9.298 | $Cit^-$ | $(H^+)_2(Cit^{3-})$ |
| 3, 0, 1 | 12.067 | Cit | $(H^+)_3(Cit^{3-})$ |
| 1, 2, 1 | 12.84 | $(H^+)(H_2VO_4^{2-})_2(Cit^{3-})$ | |
| 2, 2, 1 | 19.68 | $(H^+)_2(H_2VO_4^{2-})_2(Cit^{3-})$ | |
| 3, 2, 1 | 24.12 | $(H^+)_3(H_2VO_4^{2-})_2(Cit^{3-})$ | |
| 3, 1, 1 | 18.35 | $(H^+)_3(H_2VO_4^{2-})(Cit^{3-})$ | |
| 2, 1, 1 | 14.1 | $(H^+)_2(H_2VO_4^{2-})(Cit^{3-})$ | |
| 4, 2, 2 | 31.3 | $(H^+)_4(H_2VO_4^{2-})_2(Cit^{3-})_2$ | |
| 5, 2, 2 | 35.3 | $(H^+)_5(H_2VO_4^{2-})_2(Cit^{3-})_2$ | |
| 6, 2, 2 | 39.2 | $(H^+)_6(H_2VO_4^{2-})_2(Cit^{3-})_2$ | |

Data is taken from Ehde et al. [3]

Table 8

| (p, q, r) | log β | Formula | Extended Formula |
|---|---|---|---|
| -2, 0, 1 | -17.650 | $PO_4^{3-}$ | $[(H^+)_{-2}(H_2PO_4^-)]^{3-}$ |
| -1, 0, 1 | -6.418 | $HPO_4^{2-}$ | $[(H^+)_{-1}(H_2PO_4^-)]^{2-}$ |
| 1, 0, 1 | 1.772 | $H_3PO_4$ | $[(H^+)(H_2PO_4^-)]$ |
| 9, 14, 1 | 90.7 | $[(H^+)_9 (H_2VO_4^{2-})_{14} (H_2PO_4^-)]^{6-}$ | |
| 10, 14, 1 | 94.84 | $[(H^+)_{10} (H_2VO_4^{2-})_{14} (H_2PO_4^-)]^{5-}$ | |
| 11, 14, 1 | 96,41 | $[(H^+)_{11} (H_2VO_4^{2-})_{14} (H_2PO_4^-)]^{4-}$ | |

Data is taken from Selling et al. [4]

References


1. Alderighi, L.; Gans, P.; Ienco, A.; Peters, D.; Sabatini, A.; Vacca, A. *Coor. Chem. Rev.* **1999**, *184*, 311-318
2. Pettersson, L.; Hedman, B.; Andersson, I.; Ingri, N. *Chem. Scr.* **1983**, *22*, 254-264.
3. Ehde, P.; Andersson, I.; Pettersson, L. *Acta. Chem. Scand.* **1989**, *43*, 136-143.
4. Selling, A.; Andersson, I.; Pettersson, L.; Schramm, C.; Downey, S.; Grate, J. *Inorg. Chem.* **1994**, *33*, 3141-3150.
